# From Small Molecules toward Whole Cells Detection: Application of Electrochemical Aptasensors in Modern Medical Diagnostics

**DOI:** 10.3390/s21030724

**Published:** 2021-01-21

**Authors:** Robert Ziółkowski, Marta Jarczewska, Łukasz Górski, Elżbieta Malinowska

**Affiliations:** 1 Faculty of Chemistry, Warsaw University of Technology, Noakowskiego 3, 00-664 Warsaw, Poland; mjarczewska@ch.pw.edu.pl (M.J.); lukegor@ch.pw.edu.pl (Ł.G.); 2 Center for Advanced Materials and Technologies, Warsaw University of Technology, Poleczki 19, 02-822 Warsaw, Poland

**Keywords:** aptasensors, electrochemistry, medical diagnostics, SELEX, metal cations, cancer biomarkers, microbial toxins

## Abstract

This paper focuses on the current state of art as well as on future trends in electrochemical aptasensors application in medical diagnostics. The origin of aptamers is presented along with the description of the process known as SELEX. This is followed by the description of the broad spectrum of aptamer-based sensors for the electrochemical detection of various diagnostically relevant analytes, including metal cations, abused drugs, neurotransmitters, cancer, cardiac and coagulation biomarkers, circulating tumor cells, and viruses. We described also possible future perspectives of aptasensors development. This concerns (i) the approaches to lowering the detection limit and improvement of the electrochemical aptasensors selectivity by application of the hybrid aptamer–antibody receptor layers and/or nanomaterials; and (ii) electrochemical aptasensors integration with more advanced microfluidic devices as user-friendly medical instruments for medical diagnostic of the future.

## 1. Introduction

### 1.1. Aptamers

**Figure 1:**
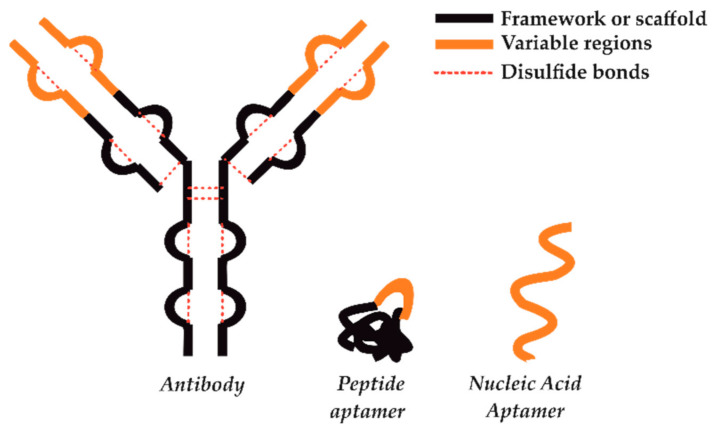
Schematic representation of antibody, peptide, and nucleic acid aptamers both with their relative sizes and possible spatial constrains [2].

The term aptamer comes from the Latin word “aptus” meaning fitting and the Greek word “meros” that means particle. It describes DNA/RNA oligonucleotides or peptides that exhibit high affinity toward a specific biomolecular species of interest [1,2]. These two classes of synthetic biomolecules, nucleic acids (NA) aptamers and peptide aptamers (PA), show a large variability in functional groups (i.e., different pH dependence on the formation of hydrogen and electrostatic interactions) and conformational flexibility [2]. In turn, this allows for the design and production of a vast number of different aptamers selective for almost any analyte of interest, from small molecules toward even whole cells [2,3]. The idea of nucleic acids (NA) aptamers originated from the Craig Tuerk experiment conducted in 1990, which related to changes in “translational operator” within the bacteriophage T4 gene 43 mRNA [1]. The introduced mutation reduced the binding loop size from eight nucleotides to four, even though it bound with the same affinities to the gene 43 protein. The winning nucleic acid sequence was one of over 65,000 investigated, and the process of the new loop selection was called SELEX [1]. The peptide aptamers (PA) were developed around 1996, and the concept was originally introduced by Roger Brent [4]. Contrary to flexible strands of nucleic acids, the PA is built with the use of small and very stable protein backbone called “scaffold”, within which a short (5–20) amino acid sequence is embedded [2]. As the conformational constraint stabilizes the insert loop and makes it more likely to fold and recognize cognate surfaces, PAs can be viewed as a scaled-down version of an antibody. However, unlike antibodies, peptide aptamers are characterized by significantly smaller size, simple design, and disulfide-independent folding, which gives them the possibility to function also inside living cells. Contrary to chemically synthesized NA, PA are selected and produced in vivo through yeast two-hybrid methods, which in turn makes them an ideal candidate for interrogating intracellular targets in a physiological environment. The other difference between NA and PA also concerns their contact surface during the interactions with the analyte of interest. Contrary to PA, NA aptamers extend the surface contact with their targets because of the possibility of adaptive conformational changes, which leads to the more precise and stronger binding. This results in a large interaction surface, similar to immunoglobulins (i.e., ≈2600 Å2) (Figure 1). In turn, PA is characterized by a smaller binding footprint; however, this permits for a more precise interrogation of the target than that offered by NA [2].

Such a highly selective molecular recognition creates the possibility of aptamers application in therapy and diagnostics [5], targeted drug delivery [6], molecular imaging [7], and biosensors [8]. However, the presented manuscript is dedicated only to the last of the above. The various colorimetric, fluorescent, quartz crystal microbalance, SPR (Surface Plasmon Resonance), and electrochemical biosensors were designed and fabricated with the aptamers as recognition elements [9,10,11,12,13]. Among these techniques, electrochemical methods are widely combined with aptamers-based target recognition due to, e.g., well-developed theory, facile design, low cost, high sensitivity, easy operation, short response times, or the simplicity of its incorporation into more advanced microfluidic diagnostic devices. Additionally, the employment of nanomaterials such as graphene oxide (GO), gold nanoparticles, and so on may further assist or improve the analysis [13,14]. As during the design of such devices, one of the crucial parameters is the possibility of the introduction of different chemical modifications to the sensing molecular probe, which allows for use of the chosen mechanism of detection without significant changes in receptor affinity; herein, only nucleic acids-based electrochemical aptasensors will be described. Moreover, one of the greatest advantage of nucleic acid aptamers is the possibility of their use in a variety of environmental conditions and the robustness and speed of its in vitro generation, selection, and evolution of aptamers within the SELEX procedure [1,2,15].

### 1.2. Procedure for Nucleic Acid Aptamer Sequence Selection—SELEX

Aptamer strands that show high affinity toward a defined analyte are obtained using the SELEX method. As was already mentioned, this technique was first introduced by two independently working research teams in 1990. Tuerk and Gold were focused on the interaction between T4 DNA polymerase (gp43) and the ribosome-binding site of miRNA that were responsible for encoding the enzyme. Through their work, it was possible to select gp43 binding sequences, and they named the process SELEX. In the meantime, a similar procedure was applied by the other team of Ellington and Szostak, who managed to isolate RNA strands that were able to form three-dimensional structures as a result of interaction with small organic dyes, including Cibacron Blue and Reactive Blue 4 [16,17,18].

**Figure 2:**
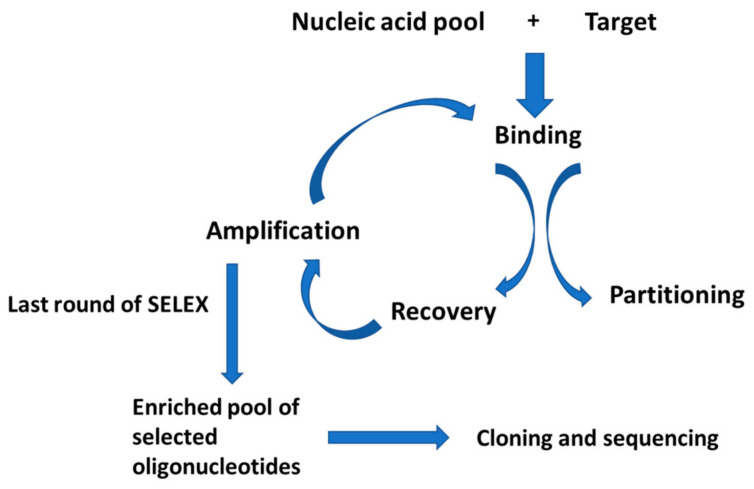
Schematic representation of the SELEX process.

The SELEX process (Figure 2) consists of repetitive cycles that allow identifying a pool of RNA or DNA strands that show the highest affinity toward a defined target analyte. The first step of that technique is the formation of a pool of RNA or DNA sequences that are obtained using combinatorial techniques, which result in a collection of strands that reaches even 10^15^ different molecules [16,17,18,19,20,21]. The aptamer strand consists of a central random region composed of 20–80 nucleotides and flanking constant sequences that contain 18–21 nucleotides that allow for PCR reaction as they bind to primer sequences [22,23,24,25,26,27]. Sometimes, the aptamers can be additionally tagged with fluorophore or radiolabel. It should be noted that in the case of the RNA library, there is a necessity for the transformation of a random DNA collection before the first cycle of the SELEX process. In such a case, a special sense primer with a 5′ end containing the T7 promoter sequence and an antisense primer are needed to change the ssDNA library into a dsDNA library. The dsDNA library is transcribed by T7 RNA polymerase and a randomized RNA library that allows for RNA SELEX. Then, the obtained library is incubated with a chosen analyte, and aptamer–analyte complexes are formed. Next, the unbound aptamer strands are separated from complexes. For that purpose, size exclusion filtration using nitrocellulose, affinity chromatography, or the magnetic beads, as well as capillary chromatography and flow cytometry, is applied. Then, the complexes are uncoupled, and aptamers are eluted by the change of ionic strength, temperature, or by the use of chaotropic agents. Next, the pool of aptamer strands become amplified using the PCR and RT PCR techniques. In the case of RNA, in each round, the sequences are reversely transcribed and amplified using the RT-PCR technique. As a result, a smaller but enriched collection of aptamer strands is obtained and used for another round of selection. In the case of RNA collection, a pool is formed by in vitro transcription. Usually, 8–15 rounds of selection are conducted, and the remaining pool of sequences is cloned and sequenced [16,19]. In this case, NMR and crystallography studies are performed to gain more information on the three-dimensional structures of obtained aptamer strands.

It should be noted that since the first use of the SELEX method, there are several examples of modified methods, including capillary electrophoresis SELEX, toggle SELEX, tailored SELEX, and Chimeric SELEX [17,28]. In the case of capillary electrophoresis SELEX, the minimization of the number of SELEX rounds was achieved and can refer to four rounds [23,28,29,30]. Toggle–SELEX is based on the selection of aptamer strands that can bind to two different analytes. In the first round, the aptamers are incubated with the first analyte and then amplified. Then, the enriched pool is incubated with the second analyte. Such a process is repeated and usually, thirteen rounds of selection are conducted [31]. Tailored SELEX allows a primer-free selection of aptamers. After each cycle of RNA strands, the strands are ligated with primers and transcribed reversely into cDNA. The reverse strand is cleaved with alkaline fission. Then, truncated strands serve as templates for transcription and the next round of selection. Chimeric SELEX enables receiving the RNA aptamers against two distinct target analytes. RNA aptamers are transcribed into cDNA and amplified. This is followed by mixing two cDNA pools, heating and annealing the primer binding regions. Those regions are extended to obtain chimeric dsDNA. DNAs are transcribed into RNA, which is reselected to enrich the population, and the activity of both aptamers is retained within one aptamer strand [28,32]. One round of SELEX was also performed to minimize the use of chemicals and safe time [18]. There have been also approaches for minimizing the number of SELEX rounds to one, and the example could be MonoLex SELEX that employs affinity chromatography that was used, e.g., for identification of the vaccinia virus [33]. Contrary to antibodies, aptamers exhibit a longer shelf life and can be regenerated by the use of proper conditions. It should be noted that most aptamers exhibit binding affinities expressed as dissociation constants in 10^−9^ M to 10^−12^ M values [16,18,23]. Furthermore, it is possible to adjust conditions for the SELEX process, and for instance, it is possible to apply nonphysiological conditions for the identification of aptamer molecules. The aptamer can be obtained through chemical synthesis with high efficiency, and it is possible to introduce several modifications into the strand to decrease its susceptibility to nucleases. Such changes can be conducted after performing the SELEX process and may refer in the case of RNA sequences to the substitution of the 2′ OH group in the ribose ring with an -NH_2_, -F, or 2′–O-methyl group [16,17,19,20,21]. The application of the SELEX method also has some limitations as it is not possible to obtain aptamer strands for all kinds of molecules. Generally, it is easier to find sequences showing high affinity toward more complex molecules, including proteins and cells, since there are several types of interactions that allow for binding between the aptamer and target analyte. On the contrary, it is hardly possible to identify aptamer strands specific to small analytes such as metal cations, and so far, sequences showing high affinity to potassium, lead, mercury, arsenite and cadmium ions were presented. Moreover, there is no standardized protocol of the SELEX process; hence, it is necessary to adjust the conditions for each type of target molecule. It should also be noted that there is a high possibility of enrichment of aptamer pool with sequences that do not exhibit high affinity toward the chosen analyte. However, it should be emphasized that nowadays, the SELEX procedure does not take as much time as it used to, and in many cases, it can last up to a few hours instead of weeks. Nevertheless, so far, only some part of the SELEX procedure was automized.

### 1.3. Advantages of Aptamers over Other Biologically Derived Receptors

Aptamers are often compared to antibodies as bioreceptors, with some of the aptamer properties being even more favorable for the development of biosensors. One of the main advantages of aptamers is the well-established SELEX process for the selection of aptamers binding given analyte. Moreover, the large-scale production of aptamers is relatively easy and economical with the use of conventional chemical methods [34]. This is in sharp contrast to antibody production, typically done in animals, which is a more expensive and less reproducible process with serious ethical issues. Aptamers show a wide range of possible targets, including simple inorganic ions, small organic compounds (antibiotics, vitamins, neurotransmitters, drugs, etc.), viruses, whole cells, as well as peptides and proteins, which seem to be the most popular targets for aptamers. Antibodies selective toward small molecules or toxic compounds are often impossible to produce, while developing aptamers for such targets poses no problems. Aptamers offer strong interactions with the targets due to conformational changes that occur during the binding process, resulting in the folding of an aptamer around the target molecule (induced-fit mechanism). Aptamer binds its target via various non-covalent, complementary interactions, including hydrogen bonds, electrostatic attraction, and van der Waals forces, resulting in complex stability similar or even better as compared to complexes formed by antibodies [35]. Change of the aptamer conformation upon interaction with the analyte molecules allows for easy detection of the binding event using various relatively simple electrochemical or optical measurement techniques. This facilitates the development of fast and inexpensive analytical devices based on colorimetric, fluorescent, or electrochemical assays. Aptamers can be relatively easy modified with various chemical groups, e.g., for further immobilization, using well-known methods of DNA functionalization developed in molecular biology. For the fabrication of electrochemical sensors, aptamer modification with thiol moieties is popular, with the further formation of self-assembled monolayers on gold electrodes. On the contrary, chemical modification and the immobilization of antibodies on electrodes often results in the blocking of antigen recognition site and, consequently, the loss of binding properties. Aptamers also show better thermal stability than antibodies, as DNA is less prone to irreversible conformational changes than peptides. This results in easier storage and a longer shelf life of aptasensors. Aptamers typically do not cause an immune response in organisms; therefore, the development of aptasensors for in vivo measurements can be envisioned.

### 1.4. Biosensors Based on Aptamer Receptors

In recent years, biosensors have become an important tool in all areas of analytical chemistry [36]. In particular, the applications of biosensors in the clinical analysis are emerging, with the glucometer being a flagship example of a device that has been extremely successful both commercially and in helping patients suffering from diabetes. However, also other biosensors found their place in the analysis of medically important analytes.

According to the IUPAC definition, a biosensor is “a self-contained integrated device, which is capable of providing specific quantitative or semi-quantitative analytical information using a biological recognition element (biochemical receptor), which is retained in direct spatial contact with a transduction element” [37]. What is quite remarkable is that the first biosensors were electrochemical devices with immobilized enzymes, including an electrode for the amperometric monitoring of glucose concentration and an ion-selective electrode modified with urease for urea determination. DNA-modified electrodes were developed significantly later, with the first examples of such devices described in 1993 [38,39]. The development of electrochemical aptasensors occurred even later, with the first report in 2005 [40].

As evident from the definition shown above, a biosensor is defined by the recognition element present within the receptor layer. The range of possible analytes is not restricted; however, typically, biologically important substances are determined using biosensors.

Aptamers, while selected and synthesized in vitro, are chemically identical to naturally occurring RNA or DNA. Therefore, sensors with aptamers present in the receptor layer are considered as biosensors, despite the fact that they do not originate from a living organism. Another important aspect of biosensor definition is a direct contact between the receptor layer and a transducer. This is accomplished by the immobilization procedure. In the case of aptamers, the most widespread methods of immobilizing aptamers include physical adsorption, covalent bonding using sequences containing thiol or disulfide groups, EDC/NHS binding, as well as affinity interactions between streptavidin and biotin [41]. The terminal phosphate group of the oligonucleotide can be also used for immobilization on HfO_2_ or ZrO_2_ surfaces. Moreover, an aptamer can be immobilized by the hybridization with short DNA strands complementary to the fragment of the aptamer that was previously bound to the thansducer.

Electrochemical aptasensors can generate electrochemical signals using either labeled or label-free configuration. Redox properties of nucleobases (mainly guanine) can be potentially but rarely used for the development of label-free DNA sensors, including aptasensors. Such a mechanism eliminates the need for modification of the nucleic acid strands, which certainly simplifies the construction of the biosensor and reduces the costs associated with it. It should be noted that such sensors can be used only once due to damage to the receptor layer inflicted during signal readout. Moreover, the measured current signal is typically quite weak and dependent on the presence of guanine in the aptamer sequence, which eliminates it from many applications. Therefore, soluble redox indicators or labels conjugated with aptamer strand are typically used to generate a current signal in aptasensors, resulting in a higher sensitivity of analyte determination. Indicators are also necessary for impedimetric measurements (EIS—electrochemical impedance spectroscopy). In this technique, changes in impedance of the receptor layer, caused by hybridization or variations in aptamer conformation, are measured.

Aptasensors that use redox indicators or labels to generate current or impedimetric signal can be broadly divided into two groups [42]:

- Sensors based on the hybridization of an aptamer with the complementary strand. Upon interaction of an aptamer sequence with the analyte, the DNA duplex is denatured, resulting in the change of current. Both “signal-on” and “signal-off” approaches can be employed with various configurations of the receptor layer. Electrochemical labels covalently attached to either of the strands can be used here, as well as redox indicators dissolved in the sample solution.
- Sensors based on changes of the aptamer configuration upon binding with an analyte. If an electrochemical label is attached to the aptamer, such conformational changes can bring it closer or further from the electrode, resulting in an increased or decreased current, respectively. Alternatively, hybridization/denaturation of the complementary fragments within the aptamer strand can be employed, resulting in changes of the interaction with the redox indicator present within the sample solution.

## 2. Analytes for Nucleic Acids Aptamers in the Modern Medical Diagnostics

The above-mentioned nucleic acids aptamers characteristics favor their application as ultra-selective bio-recognition elements in diverse biotechnology and/or medicine related applications. This includes therapeutics such as FDA-approved biomarkers including alpha-fetoprotein (AFP) [43], carcinoembryonic antigen (CEA) [44], or carbohydrate antigen 125 (CA125) [45], and potential cancer biomarkers such as mucin 1 (MUC1) [44], neuron specific enolase (NSE) [46], osteopontin [47], vascular endothelial growth factor (VEGF165) [48], platelet-derived growth factor (PDGF) [49], epidermal growth factor receptor (EGFR) [50] and interleukin-6 (IL-6) [51], targeted delivery [52], in separation techniques [53], nanotechnology [54], and as receptors in biosensors. The first biosensing application of aptamers was presented in 1996 by Davis et al., who developed an optical aptasensor (fluorescently labeled aptamers) and applied it as a detector in flow cytometry [55]. The first use of aptamers and an electrochemical detector was presented in 2004 and was dedicated to thrombin detection. The mechanism of detection was similar to classic ELISA; however, instead of antibodies (primary and secondary), two aptamer sequences were used where one of them was labeled with glucose dehydrogenase [56]. According to the authors, it was possible to selectively detect as low as 1 µM thrombin in the sample also containing other proteins. Since then, a significant number of other electrochemical aptasensors were developed, which were dedicated to various analytes. They employed different detection mechanisms, biosensing layer composition, substrates where they were formed, and utilization of nanomaterials or microfluidic devices. In turn, this resulted in a significant decrease of lower detection limits in the developed sensors, an increase of its selectivity, and/or reduction of the assay time. In the presented review, the examples (from the past five years) of such electrochemical aptasensors will be presented.

### 2.1. Ions

Ion determination is an important aspect of clinical analytics. Both the concentrations of biologically relevant ions (Na^+^, K^+^, Ca^2+^, Mg^2+^, Cl^−^) and heavy metal ions that are toxic to the body are in the area of interest. Metal ions can be determined using various spectroscopic (e.g., ICP MS, Atomic Absorption Spectroscopy), chromatographic (ion chromatography), and electrochemical (voltammetry, potentiometry) techniques. Due to the low costs, simple measurement procedures, and the possibility of in vivo measurements, electrochemical techniques have gained special recognition in recent years. The most obvious choice for ion determination seems to be potentiometry; however, the main problem, in this case, is quite high values of detection limits. With the use of ion-selective electrodes, it is possible to determine ions at the level of micromoles, which is often not sufficient in clinical analyses, especially for the determination of heavy metal ions. In such cases, it is possible to use voltammetry, as it is capable of determining much lower ion concentrations using pulsed and stripping techniques. Often, working electrodes are modified with different substances to improve the selectivity of the analysis. One possibility is to modify the electrode with an aptamer that is selective for the selected ion. There are a limited number of aptamers characterized by increased ion selectivity; therefore, there are a few publications on aptasensors for the determination of ions. They most often concern the determination of heavy metal ions.

Mercury is one of the most toxic metals, and exposure of the human body to even trace amounts of this element can cause serious damage to the cardiovascular system, nervous system, and kidneys. Therefore, the determination of mercury ions, especially in drinking water and food, is very important. The vast majority of aptasensors proposed for the determination of mercury ions take advantage of the formation of stable complexes between Hg^2+^ and two thymine bases. The T- Hg^2+^-T base pair formed in this way is even more stable than the adenine–thymine complex, and the selectivity of the described system is considerable.

An interesting example of an aptasensor for mercury ions determination is an electrode modified with a thymine-rich DNA oligonucleotide, which, after being immobilized on the electrode, adopted the configuration of a hairpin [57]. In the presence of mercury ions and a helper oligonucleotide labeled with a ferrocene molecule in the sample solution, a DNA duplex stabilized with the T-Hg^2+^-T complex is formed, in which the electroactive ferrocene molecule is located at the electrode. The resulting analytical signal is proportional to the concentration of mercury ions in the sample. Due to the low value of the detection limit, 0.0036 nM, the proposed sensor can be used for mercury analysis in drinking water.

Nanomaterials are often used to increase the sensitivity of the mercury aptasensors. In one of such examples, sulfur−nitrogen-doped ordered mesoporous carbon was deposited on the glassy carbon electrode [58]. In the next step, gold nanoparticles were added to facilitate immobilization of the aptamer containing thymine bases via an -SH linker. In the absence of Hg^2+^, the aptamer formed a hairpin structure by base pairing. However, in the presence of mercury ions, a hairpin structure opened due to the formation of T-Hg^2+^-T adducts. This allowed hybridization of the additional short oligonucleotide modified using ferrocene label with the receptor layer. A detection limit of 0.45 pM toward mercury was reported, allowing the analysis of river and tap water samples.

**Figure 3:**
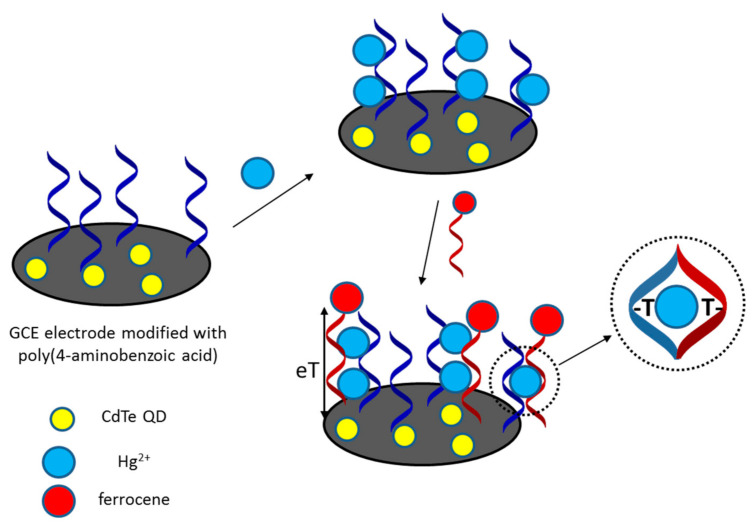
Schematic representation of the working mechanism of an aptasensors selective toward mercury cation [59].

In another design of mercury-selective aptamer, quantum dots were immobilized on a glassy carbon electrode to increase sensitivity [59]. Thymine-rich oligonucleotides formed the receptor layer, while another sequence was modified with a ferrocene label. In the presence of Hg^2+^, a complex is formed between the two DNA strands, resulting in an increase of redox current, as measured using differential pulse voltammetry. The schematic representation of the sensor working mechanism is shown in Figure 3. Developed aptasensor distinguished itself with a relatively good value of the detection limit 0.01 nM as well as selectivity against different metal ions, even at a concentration 10 times higher than that of mercury ions. Analysis of tap water samples spiked with Hg^2+^ was successfully performed.

CdTe quantum dots were also employed for the fabrication of an aptasensor based on carbon paste electrodes [60]. This transducer was coated with electrospun nanofibers of polyethersulfone containing quantum dots. A thymine-rich oligonucleotide modified with NH_2_- group was covalently attached to this electrode, and methylene blue was used as the redox indicator. The detection limit of 0.02 nM was reported for this device, with good selectivity tested over a wide range of metal cations. The determination of mercury in three types of fruit juices was successfully performed.

A relatively simple aptasensor was developed using a glassy carbon electrode modified with gold nanoflowers [61]. On this transducer, thymine-rich oligonucleotides labeled with methylene blue were immobilized using thiol chemistry. The flexibility of the ssDNA kept the redox label away from the electrode. However, in the presence of mercury ions, a hairpin structure was formed, bringing methylene blue closer to the electrode, which resulted in an increase of redox current. A remarkably low detection limit of 0.62 fM was reported for this device, and its usefulness was confirmed by the determination of mercury in milk.

A similar simple mechanism was employed in another mercury-selective aptasensor [62]. However, this time, a glassy carbon electrode was modified with reduced GO and gold nanoparticles. The thymine-rich oligonucleotide was immobilized on the electrode using an -SH moiety. This aptamer was not labeled; instead, thionine dissolved in the sample solution was used as a redox indicator. Upon the binding of a mercury ion to the oligonucleotide, it adopts a dsDNA structure, which allows the intercalative binding of thionine to the receptor layer. The current signal was measured using differential pulse voltammetry, resulting in a detection limit of 0.38 nM. The determination of mercury in lake water and tap water was successfully performed.

The same mechanism of mercury–aptamer interaction, with thionine as a redox indicator, was used in the design of another aptasensor [63]. However, this time, a magnetic glassy carbon electrode was employed as a transducer. Streptavidin-modified magnetic beads were immobilized on this electrode, and, in the next step, an aptamer modified with biotin was attached. This setup resulted in a nearly identical detection limit of 0.33 nM. Again, mercury determination in water samples was performed to prove its potential for analytical applications.

Thionine was also used in another design of an Hg^2+^ aptasensor, although this time, a much more complicated working mechanism was proposed [64]. Oriented platinum nanotube arrays, crystallized in situ on an electrode, were used as transducers for the immobilization of a thymine-containing DNA probe. On the other hand, a nanocomposite of reduced graphene oxide (rGO), Fe_3_O_4_, and thionine, modified with another oligonucleotide, was employed as a signal amplifier. In the presence of mercury ions, two DNA strands were connected via T-Hg^2+^-T pairing. A detection limit of 30 pM was reported for this device, allowing mercury determination in lake and tap water.

A somewhat similar device was named “Faraday cage-type aptasensor” [65]. A screen-printed gold electrode, used as a transducer, was modified with a thymine-containing oligonucleotide using an -SH group. Separately, a signal unit was prepared, consisting of rGO, ferrocene derivative, gold nanoparticles, and dsDNA. In the presence of a mercury ion, the signal unit was attached to the electrode via T-Hg^2+^-T complexes, resulting in an increase of current measured using fast-scan voltammetry. A detection limit of 0.03 pM was reported, allowing the determination of mercury in the tissue of an edible saltwater clam. This is an especially important application, as seafood is often a source of a considerable amount of mercury in the human diet.

While voltammetry is most often used to measure the analytical signal in mercury-selective aptasensors, other electrochemical techniques can also be employed for this purpose. An aptasensor based on electrochemical impedance spectroscopy was developed using ink-jet printed gold electrodes [66]. The thymine-rich aptamer was immobilized on these electrodes using thiol chemistry, while [Fe(CN)_6_]^3−/4−^ served as a redox indicator. The binding of mercury ions to the receptor layer resulted in an increase of RCT due to electrostatic repulsion. The reliability of the proposed device was emphasized, as well as its potential for scalability due to the controlled fabrication process.

A relatively simple aptasensor setup can sometimes result in extraordinary analytical parameters. An aptasensor was developed by the immobilization of an oligonucleotide containing no thymine bases on the gold electrode using thiol moiety [67]. Next, a partially complementary DNA strand, containing several thymines, was attached via hybridization. In the presence of a mercury ion, the second oligonucleotide folds due to the formation of T-Hg^2+^-T pairs and, as a consequence, detaches from the receptor layer. This was observed using both electrochemical impedance spectroscopy and differential pulse voltammetry, with [Fe(CN)_6_]^3−/4−^ employed as a redox indicator. The latter technique resulted in an extremely low detection limit of 0.6 zM, which is close to the detection of a single mercury ion. While this value was corrected in a corrigendum to 26 zM [68], it is still quite remarkable. The determination of mercury in municipal water and mineral water samples was successfully performed.

Lead (Pb^2+^) is one of the most toxic heavy metals, with detrimental effects on neurological, reproductive, and cardiovascular systems. It readily accumulates in human tissues; thus, the lead levels in food and drinking water should be strictly monitored. The maximum concentration of lead in drinking water is 0.07 μM; thus, sensitive analytical techniques are required to determine low levels of Pb^2+^. Numerous aptasensors were developed for this purpose. Most of them take advantage of G-quadruplexes—structures formed by guanine-rich oligonucleotides upon interaction with a metal ion. In a G-quadruplex, guanine bases form a square planar structure connected through hydrogen bonds. Such structure is also observed in thrombin binding aptamer (TBA)—possibly an aptamer that is most often used for the development of sensors; thus, lead ions interact strongly with TBA.

Many modern electrochemical Pb^2+^ aptasensors take advantage of nanomaterials to enhance the sensitivity. An example of such a device is an aptasensor using a guanine-rich DNA aptamer with a methylene blue electrochemical label immobilized by adsorption on glassy carbon electrode modified with rGO [69]. Upon the introduction of the lead ion, a G-quadruplex structure is formed, and the resulting complex dissociates from the electrode, which results in a decrease of MB current. This relatively simple setup allowed an extraordinarily low detection limit of 0.51 fM toward Pb^2+^ with good selectivity against common heavy metal ions.

An aptasensor forming the G-quadruplexes was also used as a receptor layer for electrodes with screen-printed carbon electrodes modified with a gold nanoparticles/polypyrrole composite [70]. On these transducers, an aptamer was immobilized by adsorption. In the presence of a Pb^2+^ ion, the electrochemical signal from hexacyanoferrate, used as a redox indicator, increased, allowing the determination of lead in the concentration range of 0.5–10 nM. The proposed device was successfully used to analyze lead content in human nail samples.

Graphene can also be used as an element of the redox indicator. This approach was utilized for the development of the sensor with gold electrodes modified with a guanine-rich aptamer using an -SH tag [71]. Then, graphene was attached to the receptor layer through π–π interaction, which was followed by its modification with an electroactive signaling molecule—thionine. Upon the interaction with lead ions, the conformation of aptamers in the receptor layer changes, resulting in the dissociation of graphene with adsorbed thionine, which can be observed by the diminishing of the differential pulse voltammetry current. A remarkably low detection limit of 32 fM for Pb^2+^ was reported for the proposed device, which allowed performing the determination of lead in drinking water samples.

**Figure 4:**
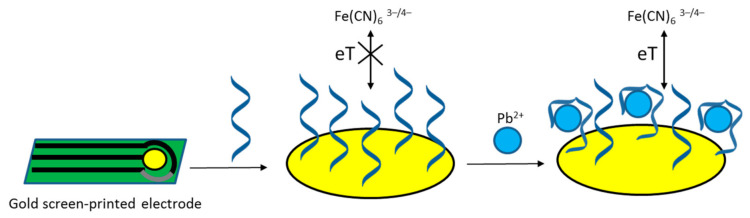
Lead determination using an electrochemical aptasensor developed on screen-printed transducers [72].

A similar approach was used for the development of another lead ion aptasensor—however, with the use of an aptamer that was selected using circular dichroism and isothermal titration calorimetry instead of the typical SELEX method [72]. This aptamer was immobilized on gold screen-printed electrodes using a polyadenine linker, and its analytical performance was studied using cyclic voltammetry and differential pulse voltammetry with hexacyanoferrate used as a redox indicator, as shown in Figure 4. The usefulness of the developed device was confirmed by the successful determination of lead in tap water and fish tissue samples.

A fairly simple Pb^2+^ aptasensor was developed by modifying screen-printed carbon electrodes with the composite of Au nanoparticles and polypyrrole [73]. Then, strands complementary to the guanine-rich aptamer were immobilized on the electrode and combined with aptamers. Toluidine blue was used as a redox indicator in this setup, resulting in high current measured using differential pulse voltammetry. In the presence of lead ions, the aptamer sequence folds into a G-quadruplex structure and detaches from the electrode, resulting in a lower current. Lead ions could be determined in the range of 0.5–25 ppb with a low detection limit of 0.6 ppb.

Nanomaterials can also be used to enhance the current response of the aptasensors by taking advantage of their catalytic properties. As an example, the use of metal–organic frameworks decorated with AgPt nanoparticles should be mentioned [74]. This material was attached to the auxiliary oligonucleotide, and it was partially complementary to the guanine-rich Pb^2+^ aptamer adsorbed on the gold electrodes. Upon the introduction of the lead ion, the aptamer folds into a G-quadruplex structure, allowing the interaction of a nanocomposite with the receptor layer. The electrocatalytic properties of metal–organic frameworks and AgPt nanoparticles result in a significant enhancement of the current signal resulted from H_2_O_2_ reduction. The developed aptasensor displayed a detection limit of 0.032 pM, allowing the determination of lead in lake and tap water.

A similar electrocatalytic system for signal enhancement was also employed in another lead aptasensor [75], although with a more complicated mechanism. Pb^2+^ ions, due to strong interaction with the guanine-rich aptamer, release the complementary oligonucleotide from the duplex, resulting in its hybridization with hairpin DNA on the electrode surface. This cycle is repeated, resulting in a large number of dsDNA on the electrode surface. Next, porous carbon loaded with platinum nanoparticles is attached using streptavidin–biotin interaction. This catalyzes the hydroquinone–H_2_O_2_ system, resulting in the measurable current signal. Such a complex mechanism of action resulted in a relatively modest detection limit toward lead ions of 18 pM—however, with good selectivity against interfering metal ions at a concentration 100 times higher than Pb^2+^.

In another work, an rGO–gold nanoparticle composite was used both as a heterogenous mediator and as an electrocatalytic label [76]. Oligonucleotides were attached to the Au nanoparticles within the nanocomposite material that was deposited on the glassy carbon electrode. Next, the same material was modified with complementary nucleotide and was subsequently immobilized on the electrode via the hybridization of both DNA strands. The current signal was measured as a result of the electrocatalytic reduction of H_2_O_2_ added to the sample. When lead ions were present in the sample solution, the nanocomposite label was detached from the electrode, resulting in a diminished current. Picomolar sensitivity of the aptasensor allowed lead determination in various samples, including lake water, tap water, and human serum.

As it can be seen from the above-described examples, lead-selective aptasensors typically use various voltammetric techniques to obtain the analytical signal. However, potentiometry can also be employed for this purpose [77]. To prepare a potentiometric Pb^2+^ aptasensor, gold electrodes were modified with gold dendrites, which were then decorated with short oligonucleotides complementary to the fragment of the guanine-rich aptamer. Separately, gold nanoparticles modified with the aptamer were prepared and then were immobilized on the electrodes via hybridization. When a lead ion-containing sample was introduced, aptamer-modified nanoparticles detach from the electrode due to aptamer folding. This results in an increase of open-circuit potential, which is measured against the Ag/AgCl electrode. A detection limit of 8.5 pM and good selectivity against other heavy metal ions allowed the determination of lead in tap, lake, and river water samples using the proposed aptasensor.

Field-effect transistors (FETs) offer a unique possibility of developing aptasensors without the use of labels or indicators. In one of such sensors, FET was modified with single-walled carbon nanotubes [78]. On this surface, dsDNA consisting of a guanine-rich lead aptamer and a complementary sequence was immobilized using terminal amino groups. Upon interaction with lead ions, the aptamer folds into a secondary structure and detaches from the transducer. This device was characterized by the detection limit of 0.39 ng·L^−1^, which allowed lead determination in spiked water samples.

In another example, FET was modified using another carbon nanomaterial: graphene [79]. Interestingly, a DNAzyme sequence was used in the receptor layer; however, it was modified to prevent its cleaving upon interaction with lead ions. The working mechanism of the proposed aptasensor is rather simple, as it relies on the change of gate potential by lead ions binding to the receptor layer. The detection limit below 37.5 ng·L^−1^ was reported for this device, and it was successfully used for the determination of lead in blood samples taken from children living in an industrial, highly polluted area.

Contamination of ground water with arsenic is an important health issue in many regions of the world. It is worth mentioning that the toxicity of this element strongly depends on its oxidation state, with As^3+^ being the most toxic. This has prompted the development of new methods for the determination of arsenite ions, including several aptasensor designs.

A relatively simple arsenite aptasensor was developed on a screen-printed carbon electrode modified with gold nanoparticles [80]. An arsenite-selective aptamer was immobilized in the next step using an -SH group. The receptor layer was further modified with polydiallyldimethylammonium cation to induce a positive charge, repelling cationic interfering substances. In the presence of arsenite in the sample solution, the aptamer was folding into a secondary structure, which resulted in an increase of current measured using differential pulse voltammetry. A detection limit of 0.15 nM was reported for this sensor, as well as remarkable selectivity against common cations, even at fivefold higher concentration. Analysis of arsenite in tap water and the lake water was successfully performed.

An interesting device was developed with the use of a field-effect transistor [81]. The gate of the FET was modified with carboxylic polypyrrole (CPPy)-coated flower-like MoS_2_ nanospheres. An aptamer modified with the amino group was immobilized covalently by the condensation with carboxylic groups of the nanomaterial. The binding of the arsenite ions to the receptor layer was directly measured as a current change caused by changing gate potential. This sensor was characterized by response time under 1 s, detection limit of 1 pM, and good selectivity. The proposed device was proven to be useful for the analysis of real samples: river water spiked with arsenite.

Electrochemical impedance spectroscopy was also employed for the development of arsenite aptasensor [82]. Glassy carbon electrode was modified with chitosan (CS)–Nafion composite. Then, glutaraldehyde was used to immobilize NH_2_-modified oligonucleotide, with a sequence complementary to the fragment of the arsenite-selective aptamer. The receptor layer was completed after immobilization of the aptamer via hybridization. The binding of arsenite to the aptamer caused its separation from the receptor layer, which was observed as an R_CT_ change. Significant signal amplification occurred when the carbon nanotubes were covalently attached to the aptamer sequence. The detection limit of 74 pM was reported for this device, allowing arsenite determination in drinking water samples.

Cadmium is one of the most toxic metals, causing serious damage to the immune, skeletal, and nervous systems, and it is also highly cancerogenic. Therefore, numerous methods for the determination of this element were developed, including electrochemical aptasensors.

A relatively simple, label-free device was developed using screen-printed gold electrodes [83]. The cadmium-binding aptamer was screened out using the isothermal titration calorimetric method. Then, the thiol-modified oligonucleotide was immobilized on the electrode, and [Fe(CN)_6_]^3−/4−^ was employed as a redox indicator for differential pulse voltammetry measurements. The detection limit of 0.05 ng mL^−1^, together with decent selectivity, allowed the determination of cadmium in water and fish tissue samples.

A methylene blue label was used for another aptasensor, with a glassy carbon electrode modified with rGO serving as a transducer [13]. After aptamer immobilization, it formed a duplex with a partially complementary DNA strand, which prevented the methylene blue (MB) label from getting close to the electrode due to dsDNA rigidness. After the binding of cadmium ion to the aptamer, the DNA duplex is separated, resulting in an increase of electrochemical signal. A remarkably low detection limit of 0.65 fM was reported for this device, which allowed the determination of cadmium in tap water samples.

An aptasensor was developed for the simultaneous determination of heavy metal ions (Hg^2+^, Cd^2+^, and As^3+^) using potentiometry [84]. A multichannel screen-printed carbon electrode array was used as a transducer, with electrodes modified with rGO and gold dendrimers. In the next step, gold nanoparticles modified with three different aptamers were deposited on corresponding electrodes. The fourth electrode, modified with a non-aptameric sequence, was used to provide an internal calibration potential. The developed device was characterized by detection limits for Hg^2+^, Cd^2+^, and As^3+^ of 2.0, 0.62, and 0.17 pM, respectively. The simultaneous determination of these heavy metal ions in samples of tap, lake, and river water was successfully performed.

Not only heavy metal ions can be detected using aptasensors. Several devices for the determination of potassium ions were also proposed. K^+^ plays an important role in the functioning of muscular, cardiovascular, and nervous systems, and it is also involved in the regulation of blood pH. The determination of potassium is crucial for the diagnosis of, e.g., heart diseases or kidney problems.

A very simple aptasensor for potassium determination was developed using a guanine-rich aptamer, which is immobilized on the gold electrode using -SH moiety [85]. In the presence of potassium ions, the aptamer forms a G-quadruplex structure, binding K^+^ ions in it. Hemin was employed as an electroactive indicator that was also bound to the G-quadruplex upon its formation caused by potassium ions. The detection limit of 0.1 nM was reported for this device; however, no real sample was analyzed.

A similar strategy, with aptamers directly tethered to a gold disk electrode via an Au-S bond, was used for the development of another potassium-selective aptasensor [86]. However, three different aptamers were tested during the research process, and it was concluded that the thrombin-binding aptamer exhibits the highest affinity toward potassium ions. Three redox indicators were compared in this study, including anthraquinone monosulphonic acid (AQMS), [Fe(CN)_6_]^3−/4−^, and methylene blue, and it was shown that the latter one contributes to the strongest electrochemical response. A detection limit of 2.31 nM and good selectivity toward K^+^ ions were achieved for the proposed sensor.

**Figure 5:**
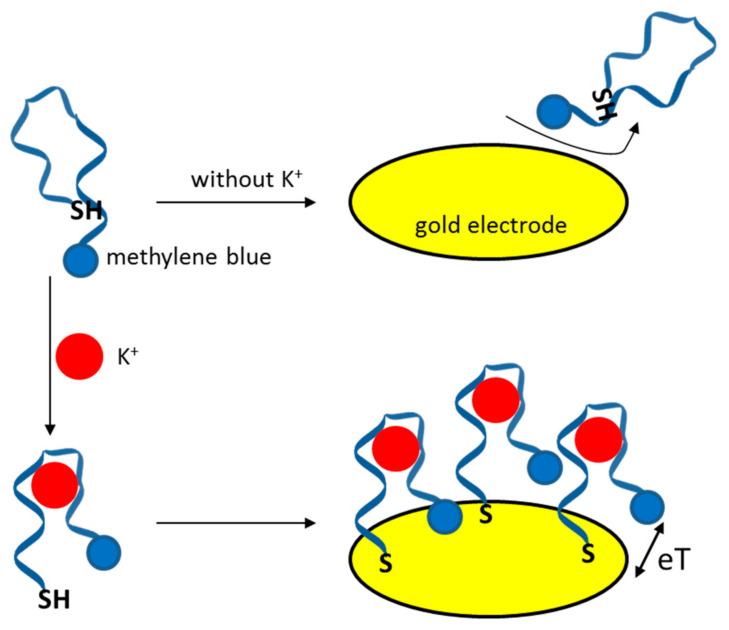
Scheme of the electrochemical aptasensor for potassium determination. Immobilization of the receptor layer on gold electrode depends on aptamer configuration, which is changed upon formation of the aptamer–K^+^ complex.

Methylene blue was also used in another aptasensor for K^+^ determination; however, this time, it was employed as a label covalently attached to a guanine-rich aptamer sequence [87]. Interestingly, the thiol group was hidden in the structure of the aptamer, and in the absence of potassium ions, the receptor layer was not formed. This is schematically shown in Figure 5. However, when potassium ions were present in the sample solution, the conformation transition was observed, and the oligonucleotide could self-assemble on a gold electrode surface. This resulted in a measurable current signal from the methylene blue label. A detection limit of 4.5 nM was reported for the developed sensor, which allowed potassium determination in lake water samples.

Electrochemical impedance spectroscopy was used for potassium determination, which employed a guanine-rich aptamer immobilized on a gold electrode by the -SH group [88]. In the presence of K^+^ ions, the formation of a G-quadruplex resulted in the conformational shift of the aptamer, which was observed as a change in the interfacial capacitance of the electrode surface. Despite a relatively high value of the detection limit, 200 nM, this device was successfully used for potassium determination in serum samples.

### 2.2. Small Chemical Compounds

As was already mentioned, the aptamers selected for small molecule targets have lower affinity than those selected for other, larger targets such as proteins [1,2,3]. This most likely results also in a smaller number of developed electrochemical aptasensors, which could be found in the literature. Nonetheless, also in this area of aptamers application, some electrochemical sensors were developed.

#### 2.2.1. Abused Drugs

##### Cocaine

**Figure 6:**
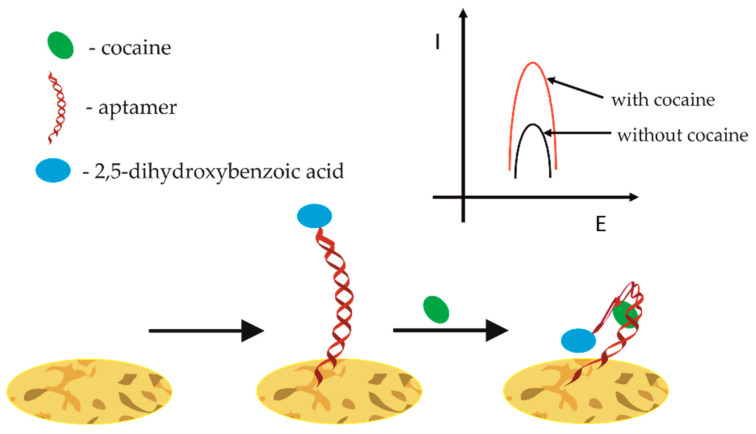
Signal on an electrochemical nanoporous gold-based aptasensor for the rapid detection of cocaine based on spatial changes of the double-stranded nucleic aptamer.

Benzoylmethylecgonine, also known as cocaine, because of its strong stimulation of the central nervous system, is one of the most popular abused drugs. Cocaine abuse causes adverse effects on humans, such as anxiety, heart failure, and organ damage. Nahid Tavakkoli et al. proposed an electrochemical aptasensor dedicated to its detection by immobilization of the 5′-disulfide-functionalized end of an aptamer sequence on a nanoporous gold (NPG) electrode followed by the conjugation of its 3′-amino-functionalized end to 2,5-dihydroxybenzoic acid (DHBA) as the redox probe shown in Figure 6 [89].

When cocaine was present in the analyzed sample, the aptamer undergoes a conformational change to a more compact, closed conformation, which reduces the distance between DHBA and the electrode surface, resulting in the enhanced electron-transfer efficiency. As the authors claim, using a square wave voltammetric method and under the optimal conditions, the linear detection response of cocaine aptasensor lies within the concentration between 0.05 and 35 mM, with a detection limit of 21 nM.

##### Codeine

Codeine (3-methylmorphine, COD), the natural opium alkaloid, is extensively consumed, either by prescription or as an illicit drug. It was used as an analgesic agent to treat mild to moderate pain and cough suppression. Although it induces less euphoria and sedation than morphine and heroin (also the same opium family), this drug abuse also is a global problem. Azadeh Azadbakht et al. proposed an electrochemical aptasensor for the sensitive detection of codeine (COD) [90]. They decorated NH_2_-functionalized Fe_3_O_4_ particles with gold nanoparticles and then placed them on a glassy carbon electrode previously modified with carbon nanotubes. Then, to such a platform, the receptors, aptamers, were covalently attached. The codeine/aptamer interaction leads to a change in the DNA conformation. In turn, according to authors, this could be transformed in the opening (unfolded aptamer) and closing (folded aptamer/codeine structure) of the gate to the long tunnels placed on the electrode surface and built with the mentioned nanomaterials. This “Off–On” strategy led to high sensitivity, simplicity, stability, and reproducibility of aptasensor. The assay has a 3.2 pM detection limit, and the electrochemical aptasensor linear response was up to 900 nM of COD.

#### 2.2.2. Neurotransmitters

**Figure 7:**
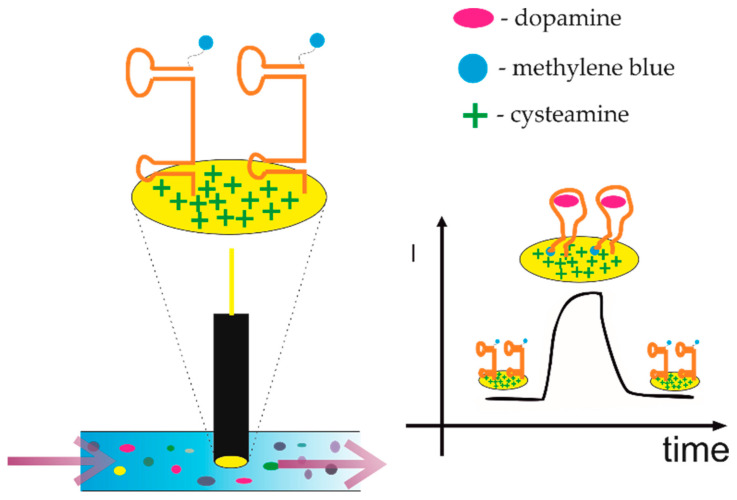
Scheme of the aptasensors construction and its integration in a wall-jet electrochemical cell with a real-time chronoamperometric response.

Neurotransmitters are essential small molecules transmitting neurological signals throughout the body and can control joy, fear, depression, insomnia, and cravings for carbohydrates, drugs, or alcohol. Changes in neurotransmitter concentrations in the human body are a characteristic of several neurological diseases (the imbalance in neurotransmitter level results in the impaired transmission of signals between neurons and other body parts). The proper diagnosis of these diseases is still a great challenge for clinicians. Among them, neurotransmitters such as dopamine, serotonin, epinephrine, or norepinephrine deserve special attention, as they play a crucial role in the development of several mental illnesses such as Alzheimer’s, Parkinson’s, Huntington’s, prion disease, multiple sclerosis, schizophrenia, migraine, depression, and so on. Despite the fact that aptamer sequences were selected for several neurotransmitters [91], the electrochemical aptasensors so far were developed only for dopamine detection. An excellent example of such is a biosensor presented by Isabel Álvarez-Martos et al. [92]. The RNA aptamer tethered to cysteamine-modified gold electrodes via the alkanethiol linker allowed for the specific electrochemical biorecognition of dopamine in human serum. This was done during continuous 20 h amperometric analysis of dopamine in 10% serum within the physiologically important 0.1–1 μM concentration range and in the presence of catechol norepinephrine and L-DOPA (dopamine precursors and metabolites, whose electrochemical “signatures” are similar to dopamine). The analysis was conducted in flow injection mode, where the aptasensor response to dopamine was within ≈1 s (almost instantly after the dopamine injection), the sensitivity 67 ± 1 nA μM−1 cm^−2^, and the dopamine lower limit of detection (LOD) was 62 nM (Figure 7). Nonetheless, despite numerous electrochemical dopamine aptasensors mentioned in the literature [92,93,94,95,96], still, the greatest challenge is its accurate measurement in body fluids. This is related to numerous neurochemical events after the dopamine (or other neurotransmitters) release, which lead to its transformation at the time-scale of a few seconds or less (the dopamine lifetime ranges from 100 to 0.05 s and depends on the current state of the organism). Thus, any neurotransmitter concentration variations could be misestimated due to the limited temporal resolution of the technique used. The same results presented in [92] still are most likely rather only a cumulative response than a time-resolved response, as the surface recognition requires time to take place.

#### 2.2.3. Antibiotic Residues

Although antibiotic detection necessity is correlated rather with food analysis, we decided to describe some examples of electrochemical aptasensors also for this class of molecules, moreover, as they are abundantly used in healthcare.

##### Streptomycin

Antimicrobial agents are usually used in bacterial infections treatment, such as tuberculosis, Mycobacterium avium complex, endocarditis, brucellosis, Burkholderia infection, plague, or tularemia. Very recently, an electrochemical aptasensor was reported for the sensitive detection of these compounds [97]. The aptasensor’s mechanism of detection utilized an arch-shaped aptamer, its complementary strand, and an enzyme, exonuclease I. The addition of streptomycin induced conformational changes between the aptamer–streptomycin structure, which resulted in the release of the complementary strand. This allowed the exonuclease to act as a digestive enzyme that degrades the free ssDNA. In turn, this lowers the steric hindrances in electrochemical indicator redox reaction on the electrode surface. As the authors claim, the developed aptasensor was highly specific for streptomycin and showed an LOD at 11.4 nM. In addition, the real sample was investigated, in the form of human serum, where an LOD as low as 15.3 nM was obtained. The aptasensor’s linear ranges for streptomycin in serum and milk were 40–1500 nM and 30–1500 nM, respectively [97].

##### Kanamycin

This aminoglycoside antibiotic has been widely used in domestic animal breeding for the prevention and treatment of infection. As a result of its overuse, kanamycin residues in animal-derived food not only cause serious side effects such as ototoxicity, nephrotoxicity, and drug allergy but also lead to increased bacterial resistance, which in the future can be a source of serious healthcare problems. To protect human health, the maximum residue limits (MRLs) for kanamycin have been established. A good example of an electrochemical aptasensor dedicated to kanamycin detection was presented by Zhou et al. [98]. The receptor layer was deposited on a gold electrode surface by Au–S chemistry with a 5′-SH-modified kanamycin-specific aptamer. The detection mechanism was related to the aptamer conformational changes, which resulted in increased electrode coverage (stem–loop structure formation after aptamer/kanamycin interactions). This lead to increased electron transfer resistance between the solution species (Fe(CN)_6_]^3−/4−^) and the same changes in the square wave voltammetry (SWV) current response. According to the authors, it was possible to selectively detect kanamycin in the range of 10–2000 nM.

##### Ciprofloxacin

Another example of antibiotic for which the European Union set the maximum residue level (MRL) is ciprofloxacin, whose concentration in milk should not exceed 100 ng mL^−1^ [99]. Ciprofloxacin (CIP) is the second generation of fluoroquinolone antibiotics and is widely used in health care. That is why its overuse in agricultural industries can lead to severe health-care problems [100,101]. Xiaobing Hu et al. used carbon nanotube (CNT)-V2O5-CS nanocomposites modified screen-printed carbon electrodes as a conductive platform for aptamer immobilization.

According to the authors, the combination of V_2_O_5_ nanoparticles biocompatibility, the efficient electron transfer capability of multiwalled CNTs, and the effective film-forming of CS allowed for the elaboration of sensitive, selective portable aptasensors on the screen-printed electrodes. Under optimal conditions, the presented dynamic response was in the range of 0.5–64.0 ng mL^−1^, with the linearity between 0.5 and 8.0 ng mL^−1^ and the LOD of 0.5 ng mL^−1^. For practical application, the operation of this aptasensor was verified in spiked milk samples where the recovery percentage of 94.50–97.87% (%RSD = 4.38, *n* = 3) was obtained.

#### 2.2.4. Other

##### ATP

**Figure 8:**
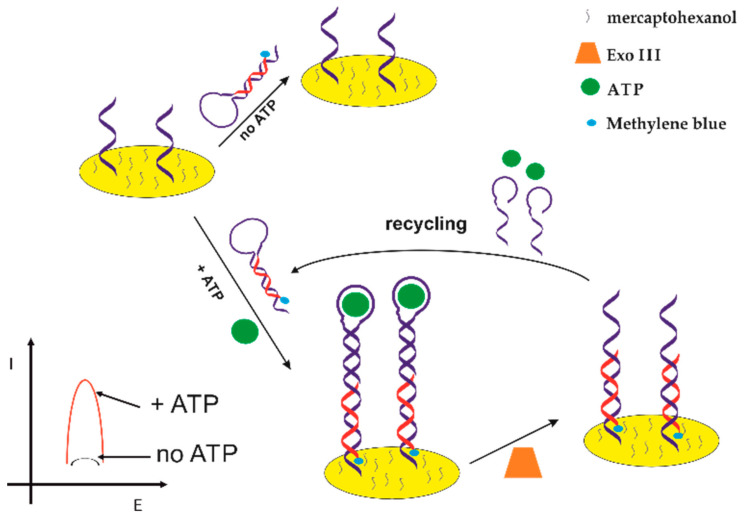
Working mechanism and construction of the electrochemical aptasensors toward adenosine triphosphate (ATP).

Adenosine triphosphate (ATP) is a multifunctional nucleoside used as an energy deposit in living cells. It plays a significant role in metabolism and many enzymatic reactions such as cellular respiration or ions transportation in cells [102,103]. The concentration change of ATP in living cells can be associated with many diseases such as angiocardiopathy or Parkinson’s diseases [104,105]. Therefore, the selective detection of ATP is of great importance in biochemical analysis and clinical diagnoses. One of the examples of the application of electrochemical aptasensors in ATP detection is a solution presented by Ting Bao et al. [106], which is shown in Figure 8.

It was based on a target-induced structure-switching and utilized exonuclease III-catalyzed target recycling for signal amplification. As can be seen in Figure 8, in the presence of ATP in the sample, methylene blue (MB) labeled hairpin DNA formed a G-quadruplex, which then led to conformational changes of the hairpin DNA and created catalytic cleavage sites for exonuclease III (Exo III). Then, the structure-switching DNA hybridized with capture DNA, which made MB close to the electrode surface. Meanwhile, Exo III selectively digested an aptamer from its 3′-end; thus, the G-quadruplex structure was destroyed, and ATP was released for target recycling. The Exo III-assisted target recycling amplified the electrochemical signal significantly. As the authors claim, under optimized conditions, the proposed aptasensor showed a linear range of 0.1–20 nM with a detection limit of 34 pM. Moreover, the sensor was characterized by good stability and selectivity.

### 2.3. Proteins and Secondary Metabolites

#### 2.3.1. Cancer Diseases Biomarkers

##### CA125

Cancer biomarker CA125 is a mucin-like glycoprotein that is recommended for clinical applications for the screening of ovarian cancer, its progression, and relapse. The normal level of CA125 is below 35 U/mL, and its elevated level can also be referred to the development of other cancers, including breast, mesothelioma as well as non-Hodgkin’s lymphoma. For its detection, a flexible FET-type aptasensor was developed where the FET transducer was modified with rGO. The aptamer strands were immobilized on the surface of carboxylated multiwalled carbon nanotubes using N-(3-dimethylaminopropyl)-N’-ethylcarbodiimide hydrochloride and N-hydroxysuccinimide chemistry. The gold electrodes were used as source and drain, whereas platinum wire was applied as a gate electrode. Poly(methyl methacrylate) (PMMA) substrate was applied for the immobilization with rGO, and this was followed by the sputtering of gold and platinum electrodes. This was followed by incubation with MWCNT/aptamer solution. The drain current decreased with the increase of CA125 molecules. It was shown that CA125 had a huge effect on the carrier mobility in the rGO channel. The proposed aptasensor showed a dynamic response toward CA125 from 10^−9^–1 U/mL (1.66 × 10^−30^ g·L^−1^ to 1.66 × 10^−21^ g·L^−1^) and a lower limit of detection of 5 × 10^−10^ U/mL (8.3 × 10^−33^ g·L^−1^). A minor response was detected in the presence of interfering molecules, including carcinoembryonic antigen (CEA), AFP, and CA15-3 [107].

##### Carcinoembryonic Antigen

CEA is a biomarker that when elevated can be associated with the development of cancer diseases, including lung, breast, and colorectal. For their detection, gold screen-printed electrodes were used that were modified with two distinct aptamer strands specific to CEA. This was followed by electrode incubation with CEA and the complementary strand to aptamer sequence. The presence of CEA led to the formation of a hairpin–loop conformation that was further bridged by a strand partially complementary to aptamer sequences. Such a construct led to an increase of steric hindrance for a [Fe(CN)6]-3/-4 redox indicator, and in the case of the presence of target analyte, a bridged construct was not formed. On the basis of differential pulse voltammetry studies, it was possible to detect CEA in the range from 3 to 40 µg·L^−1^ with a lower limit of detection of 0.9 ng·L^−1^. It was also shown that the presence of interfering molecules, including IgE, thrombin, PSA, human serum albumin (has), glycine, and myoglobin, did not cause a pronounced change of aptasensor response [108].

In another approach, CEA was detected using a lectin molecule, a concanavalin A (ConA). The gold disk electrode was modified with a thiolated aptamer and a mercaptohexanol blocking agent. This was proceeded by electrode incubation with a carcinoembryonic antigen, and this was followed by the addition of concanavalin that allowed for immobilization of the horseradish peroxidase enzyme. This enabled a catalytic reaction in the presence of hydroquinone and hydrogen peroxide. The effectiveness of each modification step was verified using cyclic voltammetry and impedance spectroscopy measurements in the presence of [Fe(CN)6]-3/-4 redox indicator. A differential pulse voltammetry (DPV) signal was derived directly from the catalytic redox reaction of hydroquinone and hydrogen peroxide. It was shown that the analytical signal increased linearly in the range from 5 to 40 µg·L^−1^ with a lower limit of detection of 3.4 µg·L^−1^. No significant change of the current response was recorded after electrode incubation with interfering molecules, including bovine serum albumin (BSA), HSA, γ-globulin, AFP, and CRP [109].

HER2 breast cancer is one of the most frequently occurring types of cancer worldwide, and one of the potential biomarkers for its detection could be human epidermal growth factor receptors (HER/erbB), which is involved in normal growth and differentiation. Malignant growth could be associated with the overexpression of human epidermal growth factor receptor 2 (HER2), which is a transmembrane tyrosine kinase receptor engaged in the development of different kinds of cancer, including breast, ovarian, gastric, and prostate. In the case of breast cancer, the HER2 level is elevated and usually reaches 14–75 ng·mL^−1^ in comparison to healthy individuals, with HER2 being at a concentration between 4 and 14 ng·mL^−1^. Hence, HER2 can serve as a potential biomarker for cancer disease. For HER2 detection, interdigitated gold microelectrodes were used that were modified with a thiolated aptamer strand. The capacitive studies revealed that it was possible to detect HER2 in the range from 1 pM to 100 nM (0.19 µg·L^−1^ to 0.019 g·L^−1^). It was also shown that the incubation of the electrode with PSA, thrombin, and HER4 did not cause a pronounced signal [110].

In another approach, a carbon working electrode was modified with a poly-l-lysine film that enabled the surface immobilization of an aptamer specific to HER2. The analytical signal referred to the current signal derived from the methylene blue redox indicator that intercalated within the double-stranded structure of aptamer. The proposed aptasensor allowed for HER2 detection in the range from 10–60 µg·L^−1^ with an LOD of 3 µg·L^−1^ [111].

HER2 was detected using a tetrahedral DNA nanostructure (TDNs), aptamer as a recognition probe, and flower-like nanozyme/horseradish peroxidase as a signal probe. A tetrahedral nanostructure was immobilized on the gold electrode surface and enabled the detection of HER2. Then, the nanozyme Mn_3_O_4_ was decorated by Pd@Pt nanozymes and linked with second aptamer and horseradish peroxidase. An Mn_3_O_4_/Pd@Pt/HRP system was applied to amplify the biosensor response by catalyzing the oxidation of hydroquinone in the presence of hydrogen peroxide. An aptamer–HER2–nanoprobe system was formed, and then a Pd@Pt/HRP/cDNA was placed on the surface of nanoprobe 1 through the hybridization of cDNA and aptamer 2, so a dendritic nanostructure was formed that amplified the signal. The proposed construct enabled HER2 detection in the range from 0.1 to 100 µg·L^−1^ with a lower limit of detection of 0.08 µg·L^−1^ [112].

In another study, an interdigitated gold microelectrode was modified with β-mercaptopropionic acid, and its carboxylic groups were activated using an EDC/NHS mixture. This allowed for surface immobilization of the aminated aptamer probe. The capacitive biosensor enabled HER2 detection in the linear range from 200 ng·L^−1^ to 2 µg·L^−1^ that was recorded at a frequency of 242 MHz. No significant change in capacitance was recorded after electrode incubation with interfering molecules, including EGFR or VEGF [113].

##### Interleukin-6

Interleukin 6 is known as a multifunctional cytokine that has an influence on the development of cancer diseases. The elevated concentration of this cytokine is happening in different types of tumor and is engaged in tumor growth and genesis, the malignant differentiation of cancer cells, immunomodulation, and metabolism. In one of the approaches, an aptasensor for interleukin 6 detection was developed using a glassy carbon electrode that was modified by electrochemical grafting of *p*-aminobenzoic acid (pABA) film. The carboxylic groups of p-ABA were activated using an EDC/NHS mixture, and this allowed for the immobilization of p-aminothiophenol (pATP). This was proceeded by tethering gold nanoparticles, which enabled the immobilization of a thiolated aptamer specific to interleukin 6. Impedance spectroscopy was utilized to monitor the effectiveness of subsequent modification steps that were used for the evaluation of aptasensor working parameters as well. It was shown that the proposed aptasensor exhibited a dynamic response toward interleukin 6 in the range from 0.005 to 100 µg·L^−1^ with the limit of detection of 1.6 ng·L^−1^. The elaborated assay showed a negligible response toward interfering proteins, including CEA, Mucin 1 (MUC1), Mucin 4 (MUC4), and Mucin 16 (Muc16) [114].

##### MUC1

MUC1 is a transmembrane glycoprotein that is considered as a potential biomarker for breast cancer diagnosis, since MUC1 is highly expressed in patients suffering from cancer disease. In one of the approaches, a competitive aptasensor was fabricated that was comprised of the cDNA-ferrocene/MXene probe. MXene (Ti_3_C_2_) was applied as a nanocarrier for the complementary cDNA–Fc probe to amplify the analytical signal. The detection procedure consisted of three processes, namely the binding of cDNA to MXene, the modification of Apt on the sensing electrode, and competitive recognition of MUC1. Glassy carbon electrode was modified with gold nanoparticles, and this allowed tethering of the thiolated aptamer. The cDNA-Fc probe was partially complementary to the aptamer strand that competes with MUC1 for binding to the aptamer. The change of aptamer conformation leads to the detachment of the Fc probe from the sensing layer, thus causing a drop in the electrochemical signal. On the basis of SWV measurements, it was shown that the aptasensor exhibited the linear range of response to the logarithm of MUC1 concentration from 1 pM to 10 µM (122 ng·L^−1^ to 1.22 g·L^−1^) [115].

In another approach, a Fluorine-doped Tin Oxide (FTO)-coated glass film was modified with carboxylated GO that was further modified with gold platinum bimetallic nanoparticles that were electrodeposited using the chronoamperometry technique. Then, the carboxylic groups of GO were activated using EDC/NHS solution. Next, streptavidin molecules were immobilized on the surface thanks to the presence of NH_2_ groups that interacted with carboxylic moieties. Finally, a biotinylated aptamer probe was immobilized on the surface. Cyclic voltammetry, differential pulse voltammetry, and impedance spectroscopy were applied for the analysis of the effectiveness of subsequent steps of electrode modification as well as of binding between aptamer and MUC1 in the presence of an [Fe(CN)6]-3/-4 redox indicator. It was shown that the proposed aptasensor exhibited a wide range of responses between 1 fM and 100 nM (122 pg·L^−1^ and 12.2 mg·L^−1^) with a limit of detection of 0.79 fM (96.4 ng·L^−1^). The elaborated aptasensor showed a smaller signal change in the presence of interfering molecules, including BSA, glucose, and MPT64 [116].

Another example focuses on the application of a receptor layer consisting of two aptamer strands that were specific to MUC1 and carcinoembryonic antigen (CEA) that contained two redox labels: methylene blue and ferrocene. For that purpose, a glassy carbon electrode was modified with gold nanoparticles, which was followed by the immobilization of aptamer probes. The prosed aptasensor showed a linear response versus the logarithm of MUC1 concentration from 5 nM to 1 µM (610 µg·L^−1^ to 0.122 g·L^−1^) with a lower limit of detection of 1.06 nM (129 µg·L^−1^). Analytical parameters were evaluated on the basis of current signals from ferrocene [117].

A methylene blue-modified aptamer probe was utilized for MUC1 detection by Karpik et al. The silicon gold-coated electrode was covered with thiolated aptamer strands. The proposed assay exhibited a response toward MUC1 in the range from 0.65 to 110 µg·L^−1^ with a lower limit of detection of 0.65 µg·L^−1^ [118].

##### PSA

Prostate cancer is concerned as one of the leading causes of cancer deaths among men. In this case, the most frequently analyzed biomarker is a prostate-specific antigen that is a glycoprotein produced by prostate epithelium. The elevated level of PSA is considered as a possibility of cancer development, and usually, the level above 4 ng·mL^−1^ is the cut-off level. For the PSA detection, a gold disk electrode was used that was modified with PSA aptamer and additionally with either mercaptohexanol or thiol-terminated sulfobetaine to minimize fouling of the electrode surface. The aptamer was tethered via coupling to the MUA layer that was activated using an EDC/NHS mixture. The binding between the aptamer layer and PSA was characterized with a K_d_ of 9.50 +/− 0.47 µg·L^−1^ [119].

**Figure 9:**
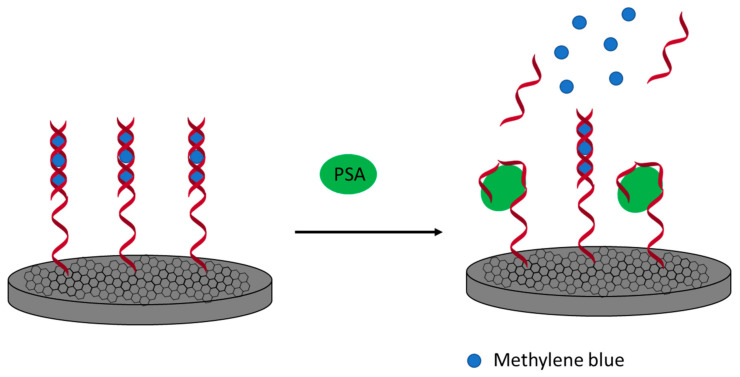
Mechanism and construction of aptasensor dedicated to PSA detection.

In another approach (Figure 9), a screen-printed electrode was modified with carboxylated GO, and carboxylic groups were activated using an EDC/NHS mixture. This allowed for the immobilization of an aminated aptamer that was specific to PSA. Finally, the aptamer-based biosensor was incubated with a complementary cDNA strand, and this enabled the intercalation of methylene blue molecules into a double strand. In the presence of PSA, a cDNA is separated from the sensing layer, and this results in a drop of methylene blue current, as there are fewer molecules bound to the receptor layer. The proposed assay allowed for PSA detection in the range from 1 ng·L^−1^ to 100 µg·L^−1^ with a lower limit of detection of 0.064 ng·L^−1^. The proposed sensor also showed a limited response toward interfering molecules such as HSA, CEA, and IgG [120].

In another approach, a molecularly imprinted polymer (MIP) polymer scaffold was elaborated for the detection of PSA. In this study, the aptamer–PSA complex was immobilized on the gold electrode surface. Then, several layers of electropolymerized polydopamine were electrodeposited. Such a procedure enabled obtaining a proper conformation of aptamer and would also enhance the binding of a prostate-specific antigen, as it would be partially entrapped. Impedance spectroscopy was used to evaluate binding between the aptamer layer and PSA. The proposed aptasensor showed a linear response in the range from 100 ng·L^−1^ to 100 µg·L^−1^. The elaborated aptasensor also exhibited a limited response toward human Kallikrein 2 protein (hK2) [121].

In another study, a glassy carbon electrode was modified with a composite of carboxylated MWCNT and GO, which was electrochemically reduced. This was followed by the electrodeposition of gold nanoparticles. Finally, the aptamer probe and mercaptohexanol were immobilized on the electrode surface. Differential pulse voltammetry and impedance spectroscopy were used to study the effectiveness of each modification step in the presence of the [Fe(CN)6]-3/-4 redox indicator. The proposed sensor showed a linear response in the range from 0.005 to 20 µg·L^−1^ with an LOD of 1 ng·L^−1^. The presence of interfering proteins such as BSA, hemoglobin, thrombin, human IgG, and lysozyme did not cause a pronounced change of the biosensor response [122].

In another approach, a triple microelectrode-based biosensor for the detection of PSA was elaborated. The microelectrodes were modified by amino-silanization on the SiO_2_ gap between the patterned gold electrodes. The aptamer strand was immobilized by the use of an EDC/NHS activation mixture. Impedance spectroscopy was applied for the analysis of the effectiveness of subsequent steps of electrode modification in the presence of an [Fe(CN)6]-3/-4 redox indicator. The proposed sensor showed a linear response in the range from 0.5 to 5000 µg·L^−1^ with a lower limit of detection of 0.423 µg·L^−1^. The obtained aptasensor exhibited a limited response toward interfering molecules: HSA and human glandular kallikrein 2 (hK2) [123].

In another study, aptamer strands specific to PSA and VEGF were immobilized on the surface of a gold-coated silicon electrode. The surface was additionally covered with mercaptohexanol to minimize nonspecific adsorption. Detection was based on the aptamer conformation change from hairpin to the unfolded strand that caused the change of location of methylene blue label present at the terminal end of aptamer probe that led to the drop of a current signal. The proposed aptasensor enabled PSA detection in the range from 0 to 100 µg·L^−1^ with a lower limit of detection of 0.08 µg·L^−1^ [124].

A gold-coated silicon electrode was modified with thiolated aptamer specific to PSA or free PSA (fPSA) that additionally contained a methylene blue label at the 3′ end that was not bound to the electrode surface. The sensor allowed for the discrimination of PSA from free PSA molecules released by human prostate cancer cells. Upon interaction with PSA, the aptamer changed its conformation, which resulted in a shift of the position of the redox label versus the electrode surface. The proposed aptasensor allowed for fPSA detection in the range from 0 to 70 µg·L^−1^ with an LOD of 2.4 µg·L^−1^. It was also possible to detect PSA when interfering molecules were present in the tested sample, including uric acid, ascorbic acid, BSA, and MUC1 [125].

Further, a glassy carbon electrode was modified with a nanocomposite composed of hemin-functionalized graphene-conjugated palladium nanoparticles, which provided high conductivity and an easily functionalized surface chemistry of palladium nanoparticles that also possessed high catalytic activity. Hemin provided a protective role as well as was used as an internal redox probe. This allowed for the surface immobilization of a biotin-based DNA probe, which was proceeded by the immobilization of streptavidin and then with an aptamer specific to PSA that was conjugated with a biotin molecule. The current signal referred to the oxidation current of hemin that increased after electrode modification with Pd nanoparticles and then decreased after incubation with DNA-biotin, streptavidin, and a biotin-based aptamer probe. Then, the current signal increased after electrode incubation with PSA. The current signal increased as the electron transfer was enhanced, and a linear response was recorded in the range from 0.025 to 204.8 µg·L^−1^ with a lower limit of detection of 8 ng·L^−1^. The current signal did not change after electrode incubation with interfering molecules, including thrombin, intravenous gamma globulin (IgG), HSA, and CEA [126].

##### VEGF_165_

The determination of cancer biomarkers is crucial for the proper diagnosis and application of the relevant treatment method. One of the potential cancer biomarkers is VEGF protein, since its overexpression is an indicator of cancer disease progression. For its detection, a glassy carbon electrode was modified with gold nanoclusters conjugated with BSA. Then, glutaraldehyde was used to further immobilize the aminated aptamer probe. For the studies on the binding between VEGF and aptamer probe, voltammetry and impedance studies were performed with the use of redox indicators: methylene blue and [Fe(CN)6]-3/-4. The binding of methylene blue to an aptamer-modified electrode led to a decrease of charge transfer resistance that further increased after electrode incubation with VEGF. On the basis of voltammetry studies, it was observed that the current changed linearly in the range from 1 to 120 pM (0.45 ng·L^−1^ to 5.4 µg·L^−1^) with a lower limit of detection of 0.32 pM (0.144 ng·L^−1^) [127].

**Figure 10:**
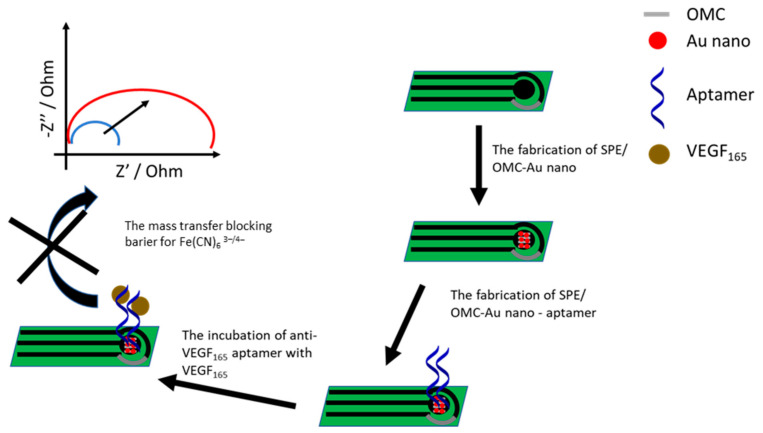
Schematic representation of construction and behavior of ordered mesoporous carbon–gold nanocomposite (OMC–Au) nano aptasensor for vascular endothelial growth factor (VEGF_165_) detection.

In another approach (Figure 10), an ordered mesoporous carbon–gold nanocomposite (OMC–Aunano) was dropped on the surface of a screen-printed working electrode. This was proceeded by the immobilization of thiolated aptamer that interacted with VEGF. The binding between the aptamer probe and protein led to the formation of steric hindrance that resulted in the increase of charge transfer resistance between the [Fe(CN)6]-3/-4 redox indicator and the electrode surface. The proposed aptasensor operated linearly in the range from 10 to 300 ng·L^−1^ with a lower limit of detection of 1 ng·L^−1^. The presence of interfering proteins human immunoglobulin G (HIgG), human immunoglobulin A (HIgA), lipase (Lip), Lysozyme (Lys), and HSA had a negligible effect on the change of biosensor response [48].

In another study, an aptamer probe specific to VEGF was immobilized on the gold surface by click reaction. The applied cathodic potential was adjusted, and the copper catalyst induced a [3 + 2] cycloaddition reaction between the alkyne-modified aptamer conjugated with a ferrocene label and the azide-functionalized electrode surface (UDTN3/UDT). The proposed aptasensor showed a response with a broad range from 0 to 10 µM (0 to 0.45 g·L^−1^) with a lower limit of detection of 6.2 nM (0.27 mg·L^−1^). The proposed sensor showed a limited response toward interfering molecules, including VEGF121, BSA, HAS, and trypsin [128].

Moreover, a capacitive hybrid aptamer–antibody biosensor was developed for VEGF detection. For that purpose, gold IDE capacitors were modified with mercaptopropionic acid, in which carboxylic groups were activated using an EDC/NHS mixture. This allowed for the surface immobilization of aminated aptamer probes. After the introduction of the target analyte, the antibodies conjugated with magnetic beads interacted with VEGF, and a sandwich structure was formed. Capacitive studies were performed in the frequency range from 50 to 300 MHz. The proposed assay showed a dynamic response toward VEGF from 5 ng·L^−1^ to 1 µg·L^−1^ [129].

#### 2.3.2. Cardiac Diseases Biomarkers

##### C-Reactive Protein (CRP)

C-reactive protein is a nonspecific biomarker of the systematic inflammatory response in the acute phase expressed principally by the liver. Hyper-sensitive CRP level is associated with the risk of occurrence of cardiovascular events in higher-risk populations, as it is concerned as one of the strongest predictors of this kind of illness. Nowadays, a turbidimetric immunoassay is typically applied for quantitative analysis of the C-reactive protein. In one of the approaches, CRP was detected using a citicoline BSA conjugate and AuNPs–aptamer nanozyme. Citicoline was coupled to BSA using a glutaraldehyde crosslinker and was coated on the 96-well ELISA plate and served as the primary antibody. Then, the aptamer conjugated with AuNPs served as a secondary binder, where AuNPs were responsible for the catalysis of the 3,3′,5,5′-Tetramethylbenzidine (TMB)/H_2_O_2_ system. AuNPs exhibited a peroxidase activity, which enabled the oxidation of TMB and allowed for the spectrophotometric detection at 652 nm. The proposed assay allowed for CRP detection in the range from 0.1–200 µg·L^−1^ with a lower limit of detection of 8 ng·L^−1^ [130].

An aptamer–antibody sandwich assay was also elaborated for CRP detection. In this system, gold nanoparticles were immobilized on the surface of a glassy carbon electrode. This was followed by incubation with an RNA aptamer specific to CRP. A secondary binder was the antibody that was tethered to the surface of SiO_2_ that was additionally covered with BSA and Zn^2+^. The analytical signal referred to the reduction signal of Zn^2+^ ions and allowed for CRP detection in the range from 0.005 to 125 µg·L^−1^ with an LOD of 0.0017 µg·L^−1^ [131].

##### Myoglobin

The serum cardiac biomarker plays an important role in clinical diagnostics, as its elevated concentration is an indication of the progression of myocardial damage. It is also the biomarker of acute myocardial infarction. The normal concentration of myoglobin in biological fluids is within the range from 0.48 to 0.9 nM or 100–200 µg·L^−1^.

For the detection of myoglobin, screen-printed electrodes were modified with rGO/carbon nanotubes dispersion. This was followed by the immobilization of the aptamer strand. The analytical signal referred to the reduction current at −0.5 V. The proposed assay exhibited a dynamic range of response from 1 µg·L^−1^ to 4 mg·L^−1^ with an LOD of 0.034 µg·L^−1^. The aptasensor also showed a limited response toward hemoglobin and BSA [132].

In another approach, an FTO electrode was modified with boron nitride nanosheets, and this was followed by the deposition of gold nanoparticles on the surface. The disulfide-based aptamer probe was incubated with a TCEP solution that enabled the reduction of a disulfide group, and this allowed for immobilization of the aptamer probe on the electrode surface. The analysis of subsequent steps of electrode modification as well as studies on the binding between aptamer receptor layer and myoglobin was performed using an [Fe(CN)6]-3/-4 redox indicator and cyclic as well as differential pulse voltammetry and impedance spectroscopy. The proposed aptasensor allowed for myoglobin detection in the range from 0.1 to 100 mg·L^−1^ with a lower limit of detection of 34.6 µg·L^−1^. It was also shown that the aptasensor exhibited a limited response toward interfering molecules, including hemoglobin, glucose oxidase, and insulin, as well as sarcosine oxidase [133].

In another study, an ITO electrode was modified with a thin film of polyethyleneimine and GO (PEI–GO). The positively charged layer of PEI-GO enabled an electrostatic attraction of the myoglobin-specific aptamer. The subsequent steps of electrode modifications were studied using an [Fe(CN)6]-3/-4 redox indicator that enabled cyclic voltammetry and impedance spectroscopy analysis. The analytical signal was referred to the reduction current of the Fe moiety present in myoglobin. A linear relation of reduction current versus the logarithm of myoglobin concentration was observed in the range from 0.001 to 1000 µg·L^−1^ with an LOD of 0.97 ng·L^−1^. Negligible responses were also recorded after electrode incubation in solutions containing interfering molecules, including cTnT, cTnI, and BSA [134].

##### Troponin

Cardiac troponin I (cTnI) is considered as one of the potential biomarkers of acute myocardial infarction. For its detection, titanium metal foil was modified with gold nanoparticles, which enabled the surface immobilization of a thiolated aptamer strand specific to troponin I. The surface was additionally modified with mercaptoethanol, which served as a blocking agent. The binding between aptamer and troponin I led to the increase of charge transfer resistance between the electrode surface and [Fe(CN)6]-3/-4 redox indicator. The proposed aptasensor showed a linear decrease of current signal versus troponin concentration between 1 and 1100 pM (0.23 ng·L^−1^ and 0.26 µg·L^−1^) with a lower limit of detection of 0.18 pM (4.3 ng·L^−1^). The aptasensor did not show a pronounced change of current signal after incubation with interfering molecules, including Chol., BSA, Mb, GOx, Ins., and IgG [135].

In another study, a magnetic metal–organic framework (MMOF) nanocatalysts and DNA nanotetrahedron (NTH) based dual-aptamer probes were applied for the detection of troponin I. Nanotetrahedron dual-aptamer capture probes were immobilized on the surface of screen-printed gold electrode. Then, MMOF Fe_3_O_4_@UiO-66 nanozymes were modified with Cu@Au nanoparticles and two kinds of the aptamer. Such a nonenzymatic nanoprobe 1 was used for the detection of cTnI that allowed amplifying the current signal by catalyzing the oxidation of hydroquinone to benzoquinone with H_2_O_2_. Nanoprobe 2 (NP2) of Cu@Au nanozymes labeled with dual complementary DNA to dual aptamer was anchored on the NP1 through DNA hybridization. This caused the formation of cluster-based nanoprobes. The proposed sensor showed a dynamic response in the range from 0.05 to 100 µg·L^−1^ with a lower limit of detection of 16 ng·L^−1^ [136].

In another approach, cardiac troponin I was detected using gold disk electrodes that were modified with thiolated aptamer strands specific to troponin I. The electrode was additionally covered with mercaptohexanol to eliminate nonspecific interaction on the surface. The analytical signal was related to ferrocene–Si nanoparticles that possessed a negative charge. In the presence of troponin I, a steric hindrance for ferrocene–Si nanoparticles was formed that led to the repulsion of nanoparticles from the electrode surface. The proposed assay allowed for troponin detection in the range from 1 to 10,000 pM (0.23 ng·L^−1^ to 0.23 mg·L^−1^) with a lower limit of detection of 1 pM (0.23 ng·L^−1^). A minor current change was recorded after electrode incubation with interfering molecules, including cTnT, cTnC, HSA, Mb, and BSA [137].

A gold electrode containing an array of nanodumbbells was applied for the immobilization of a thiolated aptamer specific to troponin I. The studies on the binding between the aptamer and troponin were performed using a [Fe(CN)6]-3/-4 redox indicator and the differential pulse voltammetry technique. The current signal decreased in the range from 0.05 to 500 µg·mL^−1^ with a lower limit of detection of 8 ng·L^−1^. The incubation with human serum albumin caused a substantial signal change, whereas the incubation with bilirubin, hemoglobin, heparin, and ethylenediaminetetraacetic acid did not cause a huge change of current signal [138].

An aptamer-based biosensor was also developed for the detection of Troponin T. For that purpose, a 40-mer aptamer was immobilized on the surface of the gold electrode, and mercaptohexanol was used to minimize the nonspecific interactions on the electrode surface. The studies on the binding between troponin and aptamer probe were conducted using a [Fe(CN)6]-3/-4 redox couple. The interaction of troponin with the aptamer-based layer led to the repulsion of the redox indicator from the electrode surface and caused the drop of the current signal. On the basis of differential pulse voltammetry, a linear response was observed that was from 0.05 to 50 µg·L^−1^ with a lower limit of detection of 23 ng·L^−1^. The proposed sensor did not show a pronounced response toward interfering molecules, including bilirubin, ethylenediaminetetraacetic acid (EDTA), hemoglobin, heparin, and HAS [139].

#### 2.3.3. Coagulation Biomarkers

##### Thrombin

Thrombin is a multifunctional protease that is formed by injured vascular endothelial cells. It is concerned as vital in coagulation cascade reactions involving anticoagulants and procoagulants as well as in the blood coagulation process. It also controls hemostasis, inflammation, and tissue adhesion. It should also be noted that it plays an important role in tumor growth and metastasis.

The aptamer specific to thrombin is one of the most studied sequences, and since its first publication in 1992, there were hundreds of examples of aptamer-based biosensors for the detection of thrombin.

In one of the approaches, a molecular recognition technology that was defined as a supramolecular noncovalent interaction between the host and guest molecules was applied for the detection of two proteins: thrombin and lysozyme. For this purpose, Dabcyl-labeled aptamer modified metal nanoparticles were prepared. An anti-thrombin aptamer was modified with a dabcyl molecule and was bound to CdS nanoparticles, whereas a lysozyme aptamer was bound to PbS nanoparticles and was also modified with dabcyl molecules. Gold electrodes were modified with thiolated β cyclodextrin that interacted with dabcyl molecules. Upon binding to the target analyte, the nanoparticle constructs were separated from the electrode surface. The electrochemical current was proportional to released nanoparticles. It was shown that the proposed aptasensor exhibited a linear response toward thrombin from 50 to 1200 pM (1.8 µg·L^−1^ to 43 µg·L^−1^) with a lower limit of detection of 17 pM (0.61 µg·L^−1^). The interfering molecules, including BSA and IgG, did not cause a pronounced change of electrochemical signal [140].

In another approach, a stem–loop aptamer was applied for the detection of thrombin that was modified with a thiol group and bisferrocene label. In the presence of thrombin, the conformation of aptamer changes, and opened conformation allowed for the binding of a thiol group to the electrode surface. The conformation changes also allowed for charge transfer between the redox label and the electrode surface. The proposed aptasensor showed a linear response in the range from 1.2 × 10^−12^ to 1.2 × 10^−8^ M (0.43 ng·L^−1^ to 0.43 mg·L^−1^) with a limit of detection of 0.8 × 10^−12^ M (0.28 ng·L^−1^). The signal intensity did not change significantly after incubation with interfering proteins, including BSA and IgG [141].

In another study, a glassy carbon electrode was modified with Nafion, which was further covered with thionin and manganese porphyrin (MnPP) catalyst. MnPP catalyzed the reaction of L-cysteine with thiol (RSH) structure to RSSR. This was followed by the introduction of nano-Au nanoparticles that enabled surface immobilization of aptamer specific to thrombin. The proposed aptasensor showed a current decrease that was linear in the range from 0 to 25 nM (0 to 0.9 mg·L^−1^) with a lower limit of detection of 0.02 nM (0.72 ng·L^−1^). No changes in the current signal were recorded when electrodes were incubated with 1 nM thrombin and 100-fold higher concentration of BSA and hemoglobin [142].

In another approach, a dual-channel strategy was applied that involved colorimetric and electrochemical strategy. In the case of colorimetric strategy, a G-quadruplex was formed that interacted with hemin that enabled the formation of DNAzyme for colorimetric detection. In the electrochemical approach, a hydroxyapatite nanoparticle was used as a signal probe and combined with magnetic nanoparticles to obtain a sandwich structure that gave an electrochemical response when thrombin was present in the solution. The system consisted of magnetic nanoparticles/thrombin aptamer 1/SP and hydroxyapatite–TBA1, hemin as well as rGO. In the presence of target analyte magnetic nanoparticles and thrombin, the aptamer bound thrombin, and this led to the release of SP. The incubation with HAP–TBA2 enabled the formation of a sandwich structure. Then, the sandwich construct was placed on the electrode surface that was modified with rGO by electrodeposition. The phosphate ions of hydroxyapatite reacted with Na_2_MoO_4_, which led to the formation of redox-active molybdophosphate precipitates that allowed for electrochemical detection. The proposed aptamer-based biosensor enabled thrombin detection in the range from 0.5 pM to 0.1 nM (0.18 ng·L^−1^ to 3.6 µg·L^−1^) with a lower limit of detection of 0.12 pM (4.32 ng·L^−1^). The elaborated sensor also showed a negligible response toward interfering molecules, including lysozyme (Lys), BSA, and L-cysteine (L-Cys) [143].

In another study, the gold electrode on a compact disk and indium tin oxide film as the opposing electrode were used. Aptamers were immobilized on the surface of the electrode along with 1-dodecanethiol as a blocking agent. [Fe(CN)6]-3/-4 was applied as a redox indicator. The proposed aptasensor showed a response toward thrombin in the range from 10 pM to 1 µM (0.36 µg·L^−1^ to 0.036 g·L^−1^) with a lower limit of detection of 10 pM (0.36 µg·L^−1^) [144].

In another approach, a host–guest recognition technology was applied. In such a case, a thrombin aptamer probe that was labeled with tetraferrocene had a double stem–loop conformation. In the absence of thrombin, the aptamer probe retained its double-stranded conformation. The steric hindrance prevented the aptamer probe to be immobilized on the electrode surface, and there was no binding between tetraferrocene and β-CD. No oxidation current of tetraferrocene was observed. When thrombin was added to the analyzed solution, it reacted with thrombin aptamer, which changed its conformation to G-quadruplex. As a result, tetraferrocene was bound to β-CD, and this caused an increase of the current signal obtained by differential pulse voltammetry. A linear, logarithmic relation between the current signal and thrombin concentration was observed from 4 pM to 12.5 nM (14 µg·L^−1^ to 0.45 mg·L^−1^). The proposed aptasensor also showed a limit of detection of 1.2 pM (0.43 ng·L^−1^). Current signals for interfering proteins, including IgG, lysozyme, and BSA, were similar to the current response for the blank sample [145].

In another study, screen–printed electrodes were modified with carbon nanofibers, and this was proceeded by the immobilization of the aptamer probe. The studies on the binding between the aptamer probe and thrombin were performed using impedance spectroscopy in the presence of the [Fe(CN)6]-3/-4 redox indicator. The aptasensor showed a linear response from 5 to 20 mg·L^−1^ with a lower limit of detection of 1.8 mg·L^−1^ [146].

In another study, the surface of the pencil graphite electrode was activated using an EDC/NHS mixture, which enabled the immobilization of an amine-based aptamer probe. The studies on the binding between aptamer and thrombin were performed using impedance spectroscopy and an [Fe(CN)6]-3/-4 redox indicator. The aptasensor showed a linear response toward thrombin in the range from 5 to 20 mg·L^−1^ with a lower limit of detection of 1.92 mg·L^−1^ [147].

**Figure 11:**
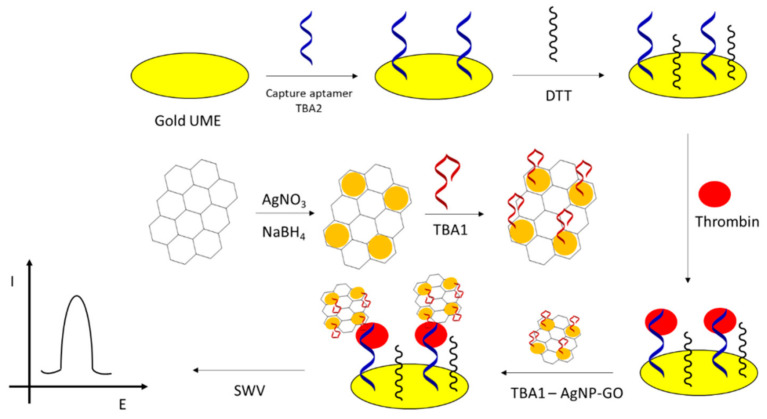
Schematic representation of sandwich aptasensor dedicated to thrombin detection.

A sandwich assay was elaborated for thrombin detection (Figure 11). The gold microelectrode was modified with a 3′ thiolated aptamer probe. After incubation with thrombin, a secondary aptamer was applied that was bound to AgNPs that decorated GO sheets. The analytical signal was related to the AgNPs presence. The oxidation current increased with thrombin concentration in the range from 0.05 to 5 nM (1.8 µg·L^−1^ to 0.18 mg·L^−1^) with a lower limit of detection of 0.03 nM (1.08 µg·L^−1^). A negligible response in the presence of interfering proteins, including AFP, CEA, and IgG, was recorded [148].

In another study, a glassy carbon electrode was modified with porous carbon nanomaterial (Z-1000), which was obtained by the carbonization of a zinc(II)-2-methylimidazole metal–organic framework. A thrombin-binding aptamer was immobilized on magnetite nanoparticles and combined with a reporter probe that was conjugated with methylene blue. When thrombin was present, thrombin aptamer/magnetite nanoparticles/thrombin was formed, which led to the separation of the reporter probe. Free molecules of the reporter probe were bound to the glassy carbon electrode thanks to the π-stacking interaction between nucleobases and the carbon nanostructure. The analytical signal was proportional to the methylene blue redox current and increased with thrombin concentration. The proposed assay showed a linear response from 10 fM to 100 nM (0.36 ng·L^−1^ to 3.6 mg·L^−1^) with a lower limit of detection of 0.8 fM (0.28 pg·L^−1^) [149].

In another approach, a glassy carbon electrode was modified with N and P co-doped GO. Then, Au nanoparticles were electrodeposited on the NP-rGO, and this was followed by the immobilization of three-dimensional DNA. After incubation with thrombin, a rolling circle amplification (RCA) reaction took place. The proposed aptasensor showed a linear response from 10^−13^ to 10^−7^ M (3.6 ng·L^−1^ to 3.6 mg·L^−1^) with a lower limit of detection of 3.53 × 10^−14^ M (1.27 ng·L^−1^). The aptasensor showed limited response toward interfering molecules, including Hb, IgG, and PSA [150].

An aptamer-sandwich construct was developed for thrombin detection. Firstly, thiolated aptamer probes were immobilized on the gold surface and further interacted with thrombin molecules. Then, a secondary aptamer interacted with thrombin that was conjugated with a composite of platinum nanoparticles and carbon nanocages. Platinum nanoparticles catalyzed the reaction of H_2_O_2_ reduction. The effectiveness of subsequent steps of the electrode modification was verified by the use of cyclic voltammetry and impedance spectroscopy in the presence of an [Fe(CN)6]-3/-4 redox indicator. The proposed aptasensor enabled thrombin detection in the range from 0.05 pM to 20 nM (1.8 ng·L^−1^ to 0.72 mg·L^−1^) with a lower limit of detection of 10 fM (0.36 ng·L^−1^). No significant change of aptasensor response was detected after electrode incubation with BSA, Hb, L-cys, and lysozyme [151].

In another approach, a graphite pencil electrode was covered with GO. Then, the electrode was incubated with a thrombin aptamer that adsorbed on the surface of GO. In the absence of thrombin, a strong guanine oxidation peak was recorded, and when thrombin was present, an aptamer–thrombin complex was separated from the electrode surface that led to a decrease of guanine oxidation current. The proposed aptasensor was characterized by a linear response in the range from 0.1 to 10 nM (3.6 µg·L^−1^ to 0.36 mg·L^−1^) with a lower limit of detection of 0.07 nM (2.52 µg·L-^1^). No change of guanine oxidation current was recorded after electrode incubation with interfering molecules, including HSA, BSA, tyrosine, phenylalanine, and hemoglobin [152].

In another study, thrombin was detected using a glassy carbon electrode that was modified with silver nanoparticles via electrodeposition, which enhanced the conductivity and increased the electrode surface area. The surface was also covered with polydopamine that was deposited on the electrode surface by self-polymerization. A thrombin aptamer was immobilized on the electrode surface by the use of the Michael addition reaction, and the surface was also covered with mercaptohexanol to minimize the nonspecific adsorption. The analysis of aptamer–thrombin binding was performed using the impedance spectroscopy technique and an [Fe(CN)6]-3/-4 redox indicator. The proposed aptasensor responded linearly toward thrombin within the logarithm of target analyte concentration from 0.1 pM to 5 nM (3.6 ng·L^−1^ to 0.18 mg·L^−1^) with a lower limit of detection of 0.036 pM (1.29 ng·L^−1^). No significant change of aptasensor response was recorded after electrode incubation with interfering molecules, including BSA, Lys, IgG, and HAS [153].

#### 2.3.4. Microbial Toxins

Toxins can be produced by bacterial, fungus, or algae and therefore are extensively present in a natural environment. Toxin contamination is unpredictable and unavoidable, which can cause severe economic losses and a public health crisis. According to the World Health Organization (WHO) [154], human beings are most likely to be exposed to toxins through the consumption of contaminated food and water. Long-term exposure to toxins can further lead to gene mutation, teratogenicity, and cancer [155].

##### Aflatoxin B

Aflatoxins are one of the most substantial mycotoxins that are present in food and animal feed. Aflatoxins are produced by molds, especially by Aspergillus flavus and Aspergillus parasiticus. Among the mycotoxins, there are four aflatoxins—B1, B2, G1, and G2—as well as two metabolites of aflatoxins M1 and M2. In the case of aflatoxin B1 (AFB1), it is present in human food and animal feed and can lead to the development of hepatic cancer, so it is recognized as a serious carcinogen. It should be noted that aflatoxin is resistant to temperature and can serve various cooking procedures. The European Union set a safe level of 2.0 mg/kg (6.4 nM) in peanuts, nuts, and other processed products. The limits for aflatoxin B1 were set between 0.05 and 20 ng·mL^−1^. For the detection of aflatoxin B1, a biosensor was constructed that consisted of a boron-doped diamond electrode that was modified with gold nanoparticles and an aptamer strand that was specific to aflatoxin B1. The electrode surface was additionally modified with 6-mercapto-1-hexanol to minimize the nonspecific interactions at the electrode surface. For the analyte quantification, the impedance spectroscopy was employed that allowed aflatoxin detection in the linear range from 10^−13^ to 10^−8^ M (31 pg·L^−1^ –3.12 µg·L^−1^) with an LOD of 5.5 × 10^−14^ M (17 pg·L^−1^). The sensor was also distinguished with specificity toward aflatoxin B1 [156].

**Figure 12:**
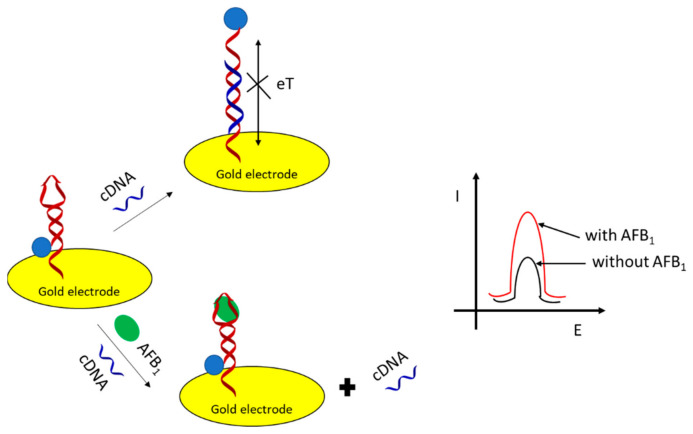
Schematic representation of the construction of aptasensor for aflatoxin B detection.

In another strategy (Figure 12), a 28-mer aptamer strand was labeled with methylene blue at the 3′ end and was immobilized on the surface of the gold electrode. In the absence of aflatoxin B1, the aptamer hybridized with complementary cDNA strand that led to the formation of a rigid double-stranded structure with redox label being in huge distance from the electrode surface that minimized the effectiveness of charge transfer between the electrode and redox label. When aflatoxin B1 was present, it competed with the complementary cDNA strand, and as a result, the aptamer formed a hairpin structure that resulted in close proximity of the redox label versus the electrode surface and enhanced the charge transfer. The proposed assay allowed for aflatoxin detection in the range from 2 nM to 4 µM (0.625 pg·L^−1^ to 1.24 mg·L^−1^), and a detection limit was 2 nM (0.625 pg·L^−1^). It should also be noted that the proposed sensor enabled rapid detection as the incubation time in the sample solution lasted for 5 min. The optimization studies provided the most appropriate length of the complementary strand as well as aptamer probe concentration and concentration of MgCl_2_ in binding solution. It was also shown that minor current change was observed in the presence of interfering mycotoxins. The proposed aptasensor was successfully used for the analysis of beer and white grape wine samples spiked with aflatoxin B [157].

The other example of aptasensor for aflatoxin B1 detection was elaborated by Nodoushan et al. In their approach, a screen-printed carbon electrode was modified with GO and gold nanowires, which was followed by the incubation with thiol-modified single-stranded DNA that hybridized with aflatoxin B1 specific aptamer strand. Then, the electrode was incubated in the solution of hematoxilin, which was the source of the analytical signal. In the presence of the target analyte, the aptamer strand formed a complex with aflatoxin B and was separated from the electrode surface. The proposed sensor showed the best performance when 0.18 mM hematoxilin was applied, and a 10 min incubation time of hematoxilin with receptor layer was used. The proposed assay allowed for aflatoxin detection in the range from 5 to 750 pM (2.19 ng·L^−1^–0.234 µg·L^−1^) with a lower limit of detection of 1.4 pM (0.44 ng·L^−1^) [158].

An aptamer-based biosensor was also applied for the determination of aflatoxin M1 that is known as the main hydroxyl metabolite of aflatoxin B1. In this approach, a glassy carbon electrode was modified with CS-modified graphene quantum dots and dendritic fibrous nanosilica functionalized by amine groups (KCC-1-NH_2_-Tb) by electrodeposition using cyclic voltammetry and chronoamperometry techniques. The aptamer strand specific to aflatoxin M1 was mixed with toluidine blue and incubated with CS-GQDs/KCC-1-NH_2_-Tb. Then, such an electrode was covered with mercaptoethanol to minimize the nonspecific interactions on the electrode surface. The obtained peak currents were symmetric with aflatoxin M1 concentration in the range from 1 fM to 0.1 uM (0.328 pg·L^−1^ to 32 µg·L^−1^), and the lower limit of quantification was 10 fM (3.28 pg·L^−1^). The proposed sensor allowed for the analysis of aflatoxin M1 in spiked milk samples and retained its stability for two days [159].

##### Staphylococcal Enterotoxin B

Staphylococcal enterotoxin B (SEB) is produced by *Staphylococcus aureus* and possess antigenic properties that result in immunosuppression and serious food poisoning by eating products such as dairy, unrefrigerated meat, and baked goods. The toxin can be transmitted through the respiratory tract and hence is concerned as a biological warfare weapon. For its detection, a carbon screen-printed electrode was applied that was modified with rGO and gold nano-urchins. This was followed by the incubation with thiolated single-stranded DNA and then with a complementary aptamer probe. [Fe(CN)6]-3/-4 was used as a redox indicator to confirm the effectiveness of subsequent modification steps. The analytical signal was referred to the current signal derived from hematoxilin that interacted with the double-stranded layer on the electrode surface. It was shown that the proposed aptasensor exhibited a linear range of response from 5 to 500 fM (0.14 ng·L^−1^ to 14 ng·L^−1^) with a lower limit of detection of 0.21 fM. (5.96 pg·L^−1^). The proposed assay did not show a pronounced change of the current signal after incubation with interfering molecules, including staphylococcal enterotoxin C (SEC), staphylococcal enterotoxin A (SEA), BSA [160].

##### Ochratoxin A

Ochratoxin A (OTA) is a mycotoxin that is secreted by Aspergillus and Penicillium that exhibits nephrotoxic, hepatoxic, neurotoxic, teratogenic, and immunotoxic activities. Ochratoxin is a contaminant that can be found in food products, including maize, rice, wheat, coffee, and dried food. For ochratoxin detection, an aminated gold electrode was applied that was modified with gold nanorods. This was followed by the electrode incubation with DNA DTN that contained an aptamer specific to ochratoxin. In the presence of ochratoxin, a g-quadruplex structure was formed that allowed the intercalation of hemin that exhibited catalytic activity toward the catalysis of PANI deposition in the presence of H_2_O_2_ that enhanced the current signal. Cyclic voltammetry and impedance spectroscopy were applied to verify the effectiveness of subsequent electrode modification steps. The proposed aptasensor showed a linear response versus the logarithm of ochratoxin concentration in the range from 0.001 to 10 µg·L^−1^ with an LOD of 0.26 ng·L^−1^. The proposed sensor showed a limited response toward interfering molecules, including aflatoxin B1 (AFB1) and zearalenone (ZEA) [161].

In another approach, gold disk electrodes were modified with thiolated complementary DNA that partially hybridized with an aptamer specific to ochratoxin A. The aptamer strand interacted with a composite that consisted of PtAg bimetallic nanoparticles and an iron–porphyrinic metal–organic framework (PCN-223-Fe). The strong electrochemical signal was derived from the oxygen reaction. The proposed aptasensor showed a wide range of response from 20 pg·L^−1^ to 2 µg·L^−1^ with LOD of 14 pg·L^−1^. The peak current did not change significantly after electrode incubation in solutions containing AFB1 and ochratoxin B (OTB) [162].

In another example, a Y-shaped nanostructure was used to detect ochratoxin A and fumonisin B. The gold electrode was modified with thiolated cDNA that interacted with an aptamer probe specific to ochratoxin A that was conjugated with gold nanorods that bind to thionin and an aptamer probe specific to fumonisin B that was bound to gold nanorods that interacted with thiolated ferrocene. In the presence of either of the target analytes, the aptamer–target molecule complex was separated from the receptor layer and resulted in a decrease of the current signal. The aptasensor allowed for ochratoxin A detection to 100 µg·L^−1^ with a lower limit of detection of 0.47 ng·L^−1^. The aptasensor showed a limited response in the presence of interfering molecules such as FB1, ZEA, and AFB1 [163].

##### Microcystins

Microcystins, produced by cyanobacteria in a water environment, belong to cyclic heptapeptide group toxins [164]. This is the most toxic and common variant among different microcystin congeners and is considered as a great threat to human health, which can lead to liver damage and even tumor promotion through the consumption of polluted food products and drinking water [165,166]. Recently, Khalil Abnous et al. developed an electrochemical aptasensor where the aptamer is immobilized on the electrode surface and hybridized with two fragments of complementary strands. Then, in the absence of microcystins, the hybridization of the aptamer to two complementary strand fragments results in an infinity-shaped DNA structure formation on the electrode surface, which repels the [Fe(CN)_6_]^3−/4−^ anions from the electrode surface. In the presence of microcystins, the double aptamer is dissociated from its complementary strand. In turn, this makes it impossible to form an infinity-shaped structure, and finally, a stronger current signal is observed. This approach allowed for the specific detection of this toxin in the range from 60 pM to 1000 nM with a detection limit of 15 pM. The developed aptasensor proved its applicability also in assays performed in real samples such as human serum [166].

##### Okadaic Acid (OA)

Okadaic acid is one of the most prevalent and largely distributed bio-toxins in the world. It is a by-product of phytoplankton, which is broadly classified under “harmful algal blooms”. The consumption of this toxin can result in nausea and diarrhea as the poisoning occurs by the inhibition of major Ser/Thr protein phosphatases—PP1, PP2A, and PP2C. Until 2011, the standard method for okadaic acid quantification was the in-vivo mouse bioassay (EC No.15/2011). As a result of the development of advanced technology and designing of selective artificial receptors, this is changing in recent years. One of the propositions for okadaic acid detection was presented in 2019 by Saipriya Ramalingam et al. [167]. The authors developed an electrochemical microfluidic biochip for the detection of OA. The screen-printed carbon electrode was modified with a phosphorene–gold nanocomposite onto which an aptamer specific to okadaic acid was immobilized. As the redox probe, the potassium [Fe(CN)6]-3/-4 was used. The microfluidic platform was designed and used to improve the reaction time as well as increase sensitivity and portability. As the detection technique, differential pulse voltammetry was applied. This allowed obtaining the detection limit at the level of 8 pM, with a linear detection range of 10–250 nM. Selectivity studies confirm the aptasensors performance in complex samples.

#### 2.3.5. Neurological Diseases Biomarkers

##### Amyloid β Protein

Alzheimer’s disease is one of the most encountered forms of dementia that causes irreversible damage to the brain that leads to memory loss and problems with thinking skills. It is said that in 2015, Alzheimer’s disease was related to 46 million cases and caused huge costs exceeding 800 billion dollars across the world. One of the possibilities of Alzheimer’s disease diagnosis is the detection of soluble Aβ oligomers, which can be considered as potential biomarker molecules as they may cause the death of neuron cells and brain disorder, e.g., by the abnormal flow of ions and synaptic dysfunction. For amyloid β protein detection, a gold disk electrode was applied that was modified with thiolated aptamer strands specific to the target analyte, and mercaptohexanol was used as a blocking agent. The subsequent steps of electrode modification, as well as binding between amyloid β and aptamer strands, were studied using impedance spectroscopy in the presence of the [Fe(CN)6]-3/-4 redox indicator. The interaction of the target analyte with aptamer-based monolayer led to the formation of a steric hindrance for redox indicator and the increase of charge transfer resistance. The biosensor response increased in the range from 0.1 to 500 nM (0.433 µg·L^−1^ to 2.165 mg·L^−1^) with a lower limit of detection of 0.03 nM (0.13 µg·L^−1^). A minor signal change was recorded in the presence of other amyloids, namely AβM and AβF [168].

##### Tau381

Tau381 is one of tau protein’s six subtypes that plays a vital role as a biomarker of early diagnostics of Alzheimer’s disease. It was found that the aptamer specific to Tau381 showed a dissociation constant of 116 +/− 6 nM. In one of the approaches, carboxylated graphite was electrostatically adsorbed on the surface of a glassy carbon electrode that was followed by the conjugation of thionin via Π–Π stacking, and then gold nanoparticles were electrodeposited on the electrode surface. The amine-based aptamer was immobilized on the gold surface thanks to Au–N interactions. The effectiveness of subsequent modification steps was verified using impedance spectroscopy in the presence of the [Fe(CN)6]-3/-4 redox indicator. The proposed aptasensor showed a linear response versus the logarithm of Tau concentration in the range from 1 to 100 pM (0.39 ng·L^−1^ to 3.97 µg·L^−1^) with a lower limit of detection of 0.7 pM (0.27 ng·L^−1^). The aptasensor exhibited a limited change in the electrochemical response in the presence of interfering molecules, including ascorbic acid, L-cysteine, glucose, and tau441 [169].

In another study, a hybrid antibody–aptamer-based construct was utilized for Tau381 detection. In such an approach, the gold electrode was modified with MPA, whose carboxylic groups were activated with EDC/NHS solution. This allowed for the immobilization of antibodies specific to Tau protein. The electrode surface was additionally covered with BSA molecules to minimize nonspecific interactions. After incubation with the target protein, the electrode was immersed in the solution containing gold nanoparticles modified with Tau-specific aptamer that was also covered with cysteamine molecules. The effectiveness of each modification step was analyzed using cyclic voltammetry and impedance spectroscopy in the presence of [Fe(CN)6]-3/-4 redox indicator. The proposed sensor responded linearly in the range from 0 to 100 pM (0 to 3.97 µgL^−1^), and the obtained lower limit of detection was 0.42 pM (0.16 ng·L^−1^). The aptasensor response toward interfering molecules such as AA, L-Cys, Glu, and tau-441 was minor [170].

### 2.4. Cells

#### Staphylococcus Aureus

**Figure 13:**
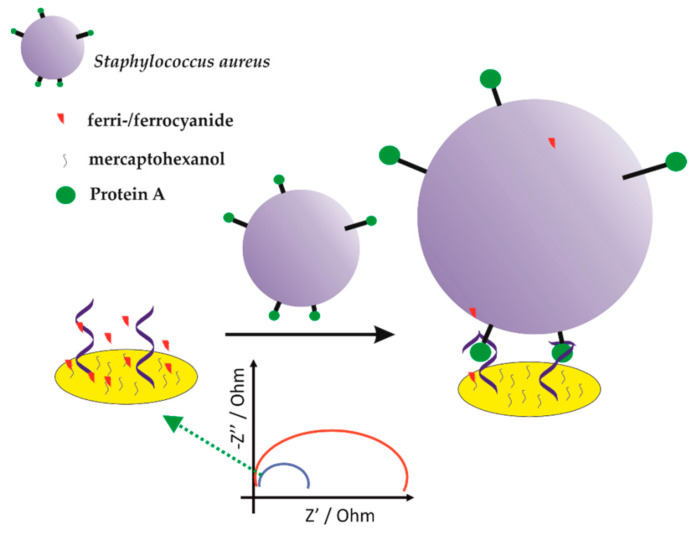
Impedimetric aptasensor scheme toward *Staphylococcus aureus detection*.

This is one of the major pathogens for humans. It causes infections, both minor ones, such as sinusitis or abscesses, and life-threatening diseases, such as bacteremia, endocarditis, and sepsis [171]. The acquired antibiotic resistance by some strains, e.g., methicillin-resistant *S. aureus* (MRSA), is a serious problem for healthcare [172]. In addition to being responsible for a number of hospital-acquired infections, *S. aureus* also produces seven different toxins that cause food poisoning [173]. In 2017, Peggy Reich et al. presented an electrochemical aptasensor, based on impedimetric measurements, dedicated to the fast and selective detection of whole cells of *Staphylococcus aureus*, as shown in Figure 13 [174].

The aptamer used was specific for protein A, which is a surface-bound virulence factor of *S. aureus*. The thiol-modified protein A-binding aptamer was immobilized onto gold electrodes by self-assembly. For passivation of the unoccupied by the aptamer electrode surface, the 6-mercapto-1-hexanol was used. The detection mechanism was based on the fact that the binding of target molecules (bacteria cells) to the immobilized aptamer decreases the electron transfer between the electrode and [Fe(CN)6]-3/-4 redox indicator present in a solution. In turn, this resulted in an impedance increase. The authors proved that upon incubation with various concentrations of *S. aureus* cells, the charge-transfer resistance increased proportionally. The obtained limit of detection was 10 CFU⋅mL^−1^, and the whole assay was performed within 10 min. The developed aptasensor was highly selective to *Staphylococcus aureus*, which was confirmed by performing the assay also versus other non-target bacteria such as *Escherichia coli* and *Staphylococcus epidermidis*.

#### H1N1

Type A influenza viruses are the most virulent and the most variable human pathogens with epidemic or even pandemic threat because of their remarkable frequency of antigenic drift and antigenic shift. That is why the rapid on-site detection of this type of virus is highly desired from the point of view of human health protection and minimizing the outbreak of infectious diseases [175]. In 2018, Chenjun Bai et al. proposed electrochemical aptasensors dedicated for whole influenza virus detection [176]. The authors presented in the paper not only the electrochemical aptasensors but also the selection of the aptamers toward inactivated influenza virus. They identified the DNA aptamers through the SELEX procedure. As the authors reported, the discriminative aptamers and their truncated sequences showed a high affinity to the inactive H1N1 virus and H3N2 virus. They also applied truncated sequences in a sandwich enzyme-linked oligonucleotide assay for an H1N1 detection, and the obtained LOD was 0.3 ng/μL. However, an electrochemical impedance aptasensor allowed lowering this value more than 300 times where the new LOD was 0.9 pg/μL, with excellent selectivity over other viruses (>100 times).

#### Circulating Tumor Cells (CTCs)

Recently, in prognostic and diagnostic approaches for cancer patients, the detection of circulating tumor cells (CTCs) has been considered as a new prognostic. This is of special importance, as early detection can significantly increase the patient’s chances. CTCs are single cells or clusters of tumor cells separated from the tumor tissue and circulate in blood and lymph node. As CTCs migration is an early event in cancer progression, CTCs detection can be critical in the early diagnosis of asymptomatic tumors [177].

#### Brest Cancer (MCF-7)

In 2018, Liang Tian et al. developed electrochemical aptasensors for the highly sensitive detection of circulating breast cancer cells [178]. The ultrasensitive electrochemical detection was obtained by the simultaneous application of rGO/gold nanoparticles composites (rGO/AuNPs composites) as an aptasensor substrate and CuO as a nanozyme. The detection of the circulating MCF-7 tumor cells was possible because of the selective recognition of the MUC-1 over-expressed on the MCF-7 cell by an MUC-1 aptamer. The CuO nanozyme was used as a signal-amplifying nanoprobe. Under the optimized experimental conditions, a wide detection range from 50 to 7 × 103 cells mL^−1^ with a detection limit as low as 27 cells mL^−1^ was obtained. The developed biosensor was also characterized by acceptable selectivity and reproducibility in human serum samples.

#### Acute Leukemia Human T-Lymphoblastic Leukemic (CCRF-CEM)

An interesting approach in acute leukemia detection was proposed by Jing Cao et al. in 2017, who, in addition to cell detection (cell line called CCRF-CEM), also proposed cell preconcentration [179]. The authors in the presented assay integrated an array of the nanochannel−ion channels with an electrochemical detection technique. The aptamer probe, selective for the protein overexpressed on the CTCs membrane, was immobilized on the mentioned ion channel surface. This allowed for the selective capturing of the CTCs. The trapped CTCs efficiently cover the ion channel entrance, which in turn leads to blocking of the ionic flow through channels. This resulted in a varied mass-transfer property of the nanochannel−ion channel hybrid. This mass-transfer change with the use of the electrochemical linear sweep voltammetry technique allowed for the sensitive detection of the CTCs. The amplified response of the array channels allowed for acute leukemia CCRF-CEM (a type of CTC) concentration and as low as 100 cells mL^−1^ could be successfully captured and detected.

### 2.5. Exosomes

Although exosomes were discovered in the 1980s, only recent works proved that they possess great potential in cancer liquid biopsy [180]. Exosomes, with a diameter of 30 to 150 nm, are recognized as extracellular vesicles that transmit molecular messengers between cells. They contribute to the growth, invasion, and metastasis of the cancer cells [181]. They are present in different body fluids, including blood, urine, cerebrospinal fluid, saliva, or breast milk, where they carry diverse molecules that are characteristic of the parent cell. They are more abundant in the samples than CTCs and much more stable. The exosomes transfer oncoproteins, RNA and DNA fragments, which provide information about the primary tumor and its environment in a minimally invasive way. It is also important that cancer cells secrete more exosomes than their non-malignant counterparts, which offers valuable information for the diagnosis. From this point of view, it is necessary to select exosome-specific surface markers that would be responsible for selective capture and detection. These can be clusters of tetraspanins (also known as the transmembrane 4 superfamily proteins), which include CD9, CD63, or CD81, integrins, or the epithelial cell adhesion molecule (EpCAM) [182].

The example of the electrochemical aptasensors dedicated to exosome detection was presented in 2019 by Li Zhao et al. [182]. The authors in their work decided to detect the specific DNA fragment (carried by exosome) after the recognition of exosomes with its aptamers. They used CD63 and EpCAM aptamers for the detection of MCF-7 cell-secreted exosomes (human breast cancer). The recognition was amplified by the application of a three-dimensional DNA walker and Exonuclease III-assisted electrochemical ratiometric sensor. Under optimal conditions, the detection limit was 1.3 × 10^4^ particles/mL with excellent selectivity. The authors also confirmed the application of the proposed assay in human serum.

## 3. Possible Development Directions of Electrochemical Aptasensors

### 3.1. Analytical Parameters Improvement

#### 3.1.1. Hybrid Aptamer–Antibody Biosensors

**Figure 14:**
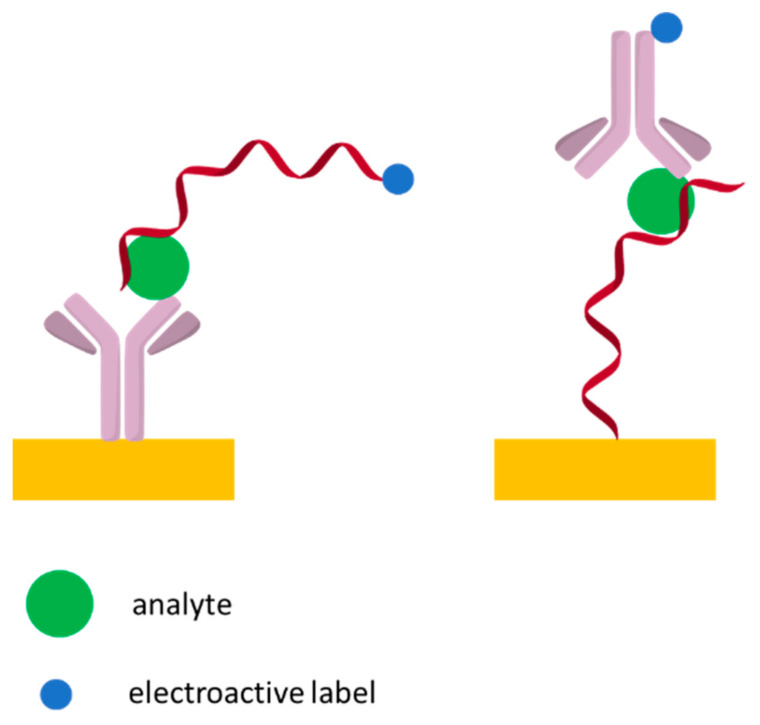
Possible configurations of aptamer–antibody hybrid receptor layers.

Since the first introduction of the aptamer sequence, there have been numerous examples of its application for the development of biosensor receptor layers, which can be utilized in clinical and environmental analysis. One of the main obstacles of receptor layers consisting solely of aptamer strands application can be high detection limits as well as limited selectivity. In the latter case, there is a high possibility of nonspecific adsorption of the components of a tested sample on the transducer surface, which might be difficult to control. That is why, recently, other approaches were proposed that refer to the elaboration of aptamer–antibody hybrid biosensors (Figure 14). In such constructs, either the aptamer or antibody can be immobilized on the transducer surface and can further interact with the target analyte. Then, a secondary binder that is again either antibody or aptamer interacts with a second binding site of the target molecule, which results in the formation of a sandwich format.

It should be noted that usually a secondary, reporter probe is conjugated with a label that typically is an enzyme or a redox indicator, which provides an electrochemical signal. As in the former case, it might catalyze a reaction that consumes or produces an electroactive compound, or in the latter case, a label possesses electroactive properties. Such constructs are often compared to classical antibody sandwich assays known as ELISA tests, and they can be called ELASA constructs (enzyme-linked aptamer sorbent assay). The main advantage of such an approach is the possibility of aptamer synthesis with high efficiency as well as simplicity of aptamer immobilization, especially on gold surfaces. Moreover, it is possible to regenerate aptamers as reporter or capture elements by the removal of a target analyte using heat or solutions of salts, acidic or basic solutions, chaotropic agents, chelating agents, or surfactants [183]. The use of hybrid antibody–aptamer layers limits the necessity of antibody production, which requires animal immunization; as in a single construct, either monoclonal or polyclonal antibodies are needed. It should be emphasized that both types of a construct (antibody–recognition probe, aptamer–reporter probe, and aptamer–recognition probe, antibody–reporter probe) can be formed on the electrode surface. Even though such an approach can be beneficial in comparison to aptamer- and antibody-based formats, there is a limited number of examples of aptamer–antibody systems. The reason for that might be the limited number of target molecules that can possess a double recognition site available for antibody and aptamer binding, which can be found mostly in proteins. Secondly, it is probable that either of the interactions between the target analyte and aptamer or antibody is stronger than the other one, which if it refers to the interaction of the reporter probe might result in the separation of a complex from the receptor layer. The other obstacle that might happen refers to the unknown manner of aptamer conformation change upon interaction with the target analyte. If an aptamer is applied as a reporter probe, its binding to the target molecule might lead to a minor conformation change that will not be recognized by the use of electrochemical methods. It is quite possible that in such a case, the position of the redox-active label that is conjugated with the aptamer probe will not change significantly after aptamer binding to the target molecule. That is why it is necessary to study more in-depth the possible aptamer conformation changes. The other obstacle that might happen in the case of antibody–aptamer hybrid layers is the necessity of the proper orientation of antibodies if they are used as recognition probes, as random antibody orientation might limit the efficient binding to the target analyte, and further formation of the sandwich construct will not be possible. Antibody immobilization might be more tedious in comparison to aptamer tethering to the surface; hence, it has to be considered, throughout, which of the molecules should be applied as a recognition and reporter probe. So far, there are several examples of aptamer–antibody sandwich constructs that refer to the following target proteins: C-reactive protein, IgE, mucin protein, platelet-derived growth factor, thrombin, and VEGF. There are also examples of hybrid systems for the detection of antibiotics, bacteria, and fungi.

#### 3.1.2. Examples of Nanomaterials Used in Aptasensor Construction

In the nearest future, electrochemical aptasensors could change the field of medical diagnostics, wherein in the modern world, ubiquitous digitalization seems to be a common goal of many fields of science and technology development [184]. As was already presented in this review, the aptamers’ versatility as molecular receptors (detection of pathogens, biomolecules, small molecules, and ions) together with their physicochemical properties as a nucleic acid (ease of synthesis and functionalization) makes them very interesting molecules in the construction of recognition layers of such digitized devices. Before developing such for commercial purposes, it is important to establish the analytical task and the specification requirements (e.g., sampling, sample preparation, analyte type, and its concentration to be detected, sample matrix) and at the same time the cost of the device. Typically, electrochemical biosensors are intended to be small and to be deployed in the field where samples cannot be processed as accurately as in a central laboratory. That is why a good electrochemical affinity-based biosensor (aptasensor) has to be developed with the use of a conductive physicochemical transducer that is able to selectively distinguish minute changes in receptor layer composition [184]. The sensitivity and selectivity of electrochemical aptasensors have been significantly improved by the application of nanotechnology and nanomaterials in their construction [134,148,150,151,152,161,162,179,182,185,186,187,188]. Nanomaterials or their composites were used as transducers for aptamer biosensing or as a label, which resulted in a significant improvement in the sensitivity by both increasing the electrochemical signal and simultaneously decreasing background noise (improved signal-to-noise ratio, S/N). It is worth mentioning that the nanocomposites can integrate the advantages of their components, which significantly improve the detection performance. Nanomaterials/nanocomposites typically can also be characterized with a large ratio of surface area to its volume with the additional number of various functional groups, which can be used for the conjugation of aptamers or selected targets. The best example of such are gold nanoparticles (AuNPs) and their composites. They are still very popular in the electrochemical aptasensors development because of their unique physical and chemical properties, such as a large surface area, a strong plasmonic characteristic, and easy functionalization with the use of self-organized monolayers [13,49,62,73,95,148,158,184,185,186]. This allowed for aptasensors development with excellent analytical performances and the cost-effective detection of various types of analytes in different types of matrixes with the use of conventional electrochemical measurement techniques such as cyclic voltammetry (CV), DPV, square wave voltammetry (SWV).

Carbon materials also are extensively used in electrochemical aptasensors development. The obtained structures (often in combination with gold nanoparticles) used as transducers outperform the traditional electrodes. This originates in the most part from structural polymorphism and chemical stability, which improve the electrochemical parameters [13,51,62,64,69,134,148,158]. In the case of graphene and its derivatives, their electrical properties can be carefully adjusted by the use of an appropriate number of stacked layers, their defect, and the presence of impurities, functional groups, or dopants, which are directly determined by the graphene production procedure [189,190]. Graphene and graphene derivatives’ unique properties of its large surface area also offer well-defined interactions with unmodified nucleic acids, which result in the high loading of strongly adsorbed single-stranded oligonucleotides such as unfolded aptamers due to π–π interactions between the nucleic bases and graphene. This adsorption is minimalized for double-stranded oligonucleotides or folded aptamers, i.e., that are bound to their target analyte. All of the above graphene properties allow for the development of a series of electrochemical aptasensors of various aptasensing schemes, and because of the separation properties, also a GO-based SELEX procedure, GO-SELEX, was successively elaborated [13,51,62,64,69,134,148,158,189,190].

### 3.2. Microfluidic Devices and Point-of-Care Testing

Recently, significant attention is given to the development of portable and highly versatile devices dedicated to complex sample analysis outside the laboratories. One of the possible, and still more and more popular, is the use of electrochemical aptasensors as detection elements in such. This is especially driven by the microelectronics progress and digitalization of the surrounded world, which led, e.g., to the production of miniaturized handheld point-of-care (PoC) devices [191,192,193,194,195]. Such a medical diagnostic development is of special interest as in an aging society, improvement of quality of life is one of the most important goals. The handheld devices enable a quick, precise, and mobile analysis of biomarkers, pathogens, or pollutants. In turn, this would significantly increase the awareness of the current health status of an individual person, and the same time, it would reduce the time necessary to implement appropriate medical procedures. Such devices, in comparison to traditional laboratory procedures, offer significantly reduced sample volume and shorter detection time, improved sensitivity due to high surface to volume ratio (also surface modified with carefully chosen nanomaterials), high throughput by parallel operation, portability, and disposability and what is no less important also real-time detection and an automated measurement process [191,192,193,194,195].

To date, such devices, where the electrochemical aptasensors are used as detection elements, were developed for various kinds of analytes. This is limited only by the sensor specificity and offers, in turn, even complete procedure automatization. One of the examples is the detection of human pathogens. Such an analysis in this case is highly desired from the point of view of the microbial agent spread (portable devices that can be used outside the laboratories) and human health and life. On-site sample processing and the detection of, e.g., the viral clinical samples are complicated, and there is a need to perform multiple complicated steps. In 2017, Rohit Chand et al. proposed a microfluidic device capable of a one-step detection of norovirus [196]. This microbial agent is extremely contagious and is the cause of more than 90% of reported non-bacterial gastroenteritis outbreaks in the United States [197]. As noroviruses are characterized with an extremely low dose of infection (<100 virions) and are fairly resistant to heating and common disinfectants [198], the user contact with the clinical sample should be reduced to the minimum. In the case of the classic detection of viral pathogens, sample purification and enrichment from the complex background often play an important role. During that and other procedure steps of sample processing, a lot of human involvement is required, which in turn can lead to an increased risk of infection among health professionals [196,197,198]. The authors [196] proposed the electrochemical sensing of norovirus using a receptor layer based on commercially available aptamer that was integrated into a disposable microfluidic platform. The microfluidic part of the device encompasses microbeads microfiltration zone and graphene/gold nanoparticle composite as a substrate for the receptor layer immobilization. The silica microbeads were used to filter out solid and larger particles from the sample solution. The composite substrate deposited at the screen-printed carbon electrode surface secured both a durable substrate for aptamer immobilization and significantly increased electron transfer rate at the interface. The mechanism of detection was based on the ferrocene tagged aptamer spatial changes, which were related to the Norovirus GII presence in the sample and the registered changes in current intensities. The authors, as a detection technique, used differential pulse voltammetry and obtained a limit of detection at the level of 100 pM for recombinant norovirus-like particles, with a detection range from 100 pM to 3.5 nM. The developed device proved its applicability in detecting norovirus in spiked blood samples also by discriminating samples containing target norovirus and other interfering molecules.

**Figure 15:**
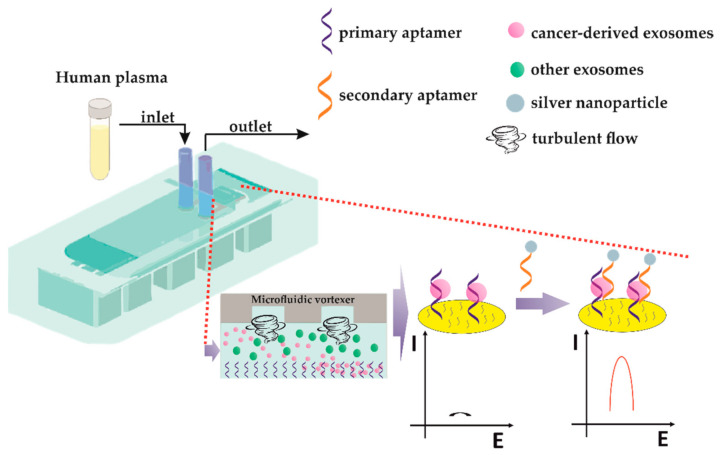
Scheme of the cancerous exosomes’ analysis with the use of dedicated microflow chip integrated with electrochemical aptasensors.

Another application of such is an approach proposed by Leila Kashefi-Kheyrabadi et al. dedicated to the sequential analysis of cancerous exosomes, as shown in Figure 15 [199].

As it was already stated, the quantification of cancer-derived exosomes has a strong potential for minimally invasive diagnosis of cancer during its initial stage. However, the significant obstacle in common cancer diagnostics with the use of exosomes is that their absolute number is still minimal to be selectively identified in a simple with efficient manner. Nonetheless, the electrochemical biosensors, which are sensitive to changes that selectively took place at their surface with additionally increased such phenomena by the application of carefully selected nanomaterials, integrated with precise fluid control in a short period of time, into more advanced miniaturized microflow device can constitute an innovative analytical platform with high potential of application in daily life use (Figure 15). In this case, the elaborated analytical platform integrated a nanostructured electrochemical aptasensor for exosome quantification, a microfluidic vortexer for continuous exosome enrichment, and a detachable assembly/disassembly unit to integrate the aptasensor and microfluidic channel for the sample injection without leakage as well as downstream analysis. The nanostructured electrochemical aptasensor was prepared by a nanocomposite consisting of MoS_2_ nanosheets (MoS2NS), graphene nanoplatelets (GNP), and CS. Such a nanomaterial combination was carefully chosen, as MoS_2_NS is an ideal substrate for the precise electrodeposition of metal nanostructures. GNP offers high electrical conductivity and an extra-large surface area. This improved the aptamers immobilization efficiency and allowed for efficient exosome capture. The sensing surface biocompatibility was secured by the application of CS. The cancerous exosomes were captured from the entire volume of exosomes. The selectivity was ensured by the immobilization on the above-described sensor surface of the epithelial cell adhesion molecule (EpCAM) aptamer. This protein is expressed on the cancer cell surface. As the cancer cell-derived exosomes are generated by inward cell budding, they also possess the EpCAM on their surface (such exosomes can be used as an indicator of cancer stages). The efficiency of the exosome’s accumulation by its capturing by aptamers was secured by specially designed geometry of microfluidic channel. Inside such a channel, the micro-vortexes are generated under laminar flow conditions. This leads to exosomes collision with the sensing surface and at the same the increase in the aptasensor sensitivity. Such an approach allows for fast enrichment of the exosomes onto the aptasensor surface, which lowers the sensing surface fouling as well as the sample volume consumption, which is especially important in the case of electrochemical sensors. The registered signal depends on the second aptamer accumulation, this time labeled with a redox probe, which results in electro-oxidation in the electrochemical signal. Such an approach allowed obtaining ultra-low LOD (17 exosomes/μL) and a low limit of quantification (100 exosomes/μL) together with a wide linearity range (1 × 10^2^ to 1 × 10^9^ exosomes/μL) in only 50 min of analysis. What is more, authors in their manuscript also proposed complete diagnosis procedures, which potentially can be incorporated into the currently valid medical standards [199].

## 4. Conclusions

The review focuses on the presentation of the latest achievements in the field of electrochemical aptamer-based biosensors. The paper covers the foundations of the SELEX process that allows for aptamer strand identification and depicts the advantages and limitations of the application of such molecules as sensing elements. Since aptamers can bind to various types of target analytes, a thorough description of recent examples of aptasensors for the analysis of metal cations, small compounds, such as neurotransmitters, protein biomarkers of cancer, cardiac and coagulation diseases, as well as bacteria and viruses cells and exomes are shown. It should be noted that a vast number of proposed aptasensors contain receptor layers that are comprised of various nanomaterials, which allow for the improvement of working parameters such as sensitivity and limit of detection. It should be emphasized that novel aptasensors can also be composed of hybrid aptamer–antibody layers that enable the improvement of receptor layer selectivity. Finally, the possibility of minimization of chemicals and sample consumption as well as the improvement of aptasensor sensitivity can be achieved by the use of microfluidic systems. It can be expected that the number of examples of aptamer-based diagnostic devices will increase as sequences for novel analytes are constantly identified by the SELEX process, and the existing aptamer–target analyte systems are adjusted, as was shown, mostly in terms of conjugation with nanomaterials and microfluidic devices.

## Data Availability

Not applicable.
